# Benchmarking Open-Source Vision–Language Models for Food Image Recognition and Portion Size Estimation

**DOI:** 10.3390/nu18213582

**Published:** 2026-10-29

**Authors:** Tommaso Marchetti, Stefano Capezzone, Rosita Esposito, Marco De Spirito, Giuseppe Maulucci

**Affiliations:** 1 Dipartimento di Neuroscienze, Sezione di Biofisica, Università Cattolica del Sacro Cuore, 00168 Rome, Italy; tommaso.marchetti@unicatt.it (T.M.); marco.despirito@unicatt.it (M.D.S.); 2 UOC Fisica per le Scienze della Vita, Fondazione Policlinico Universitario Agostino Gemelli, IRCCS (Istituto di Ricovero e Cura a Carattere Scientifico), 00168 Rome, Italy; 3 Gruppo Fastal Blu Sistemi, Via Nomentana 263, 00161 Rome, Italy; stefano@capezzone.it; 4 Digital Innovation Hub Roma, Chirale S.r.l., Via Ignazio Persico 32-46, 00154 Rome, Italy; rosita.esposito@chirale.it

**Keywords:** food images, dietary assessment, Vision–Language Models

## Abstract

**Background/Objectives**: Accurate recognition of food items and estimation of portion sizes from images is a key challenge in advancing dietary assessment and nutritional monitoring. In this study, we benchmark twenty-six configurations (seventeen model releases) of state-of-the-art open-source Vision–Language Models (VLMs), varying in size (≤15 GB) and number of parameters (<25B), on real-world food photographs collected in a university canteen, doing so to evaluate their potential for supporting automated nutrition logging and health interventions. **Methods**: We construct a curated benchmark dataset with human-annotated labels, including both food item names and scale-measured portion sizes, and assess model predictions based on recognition performance and mean absolute error in the portion estimate. The semantic matcher used to score food recognition is validated against a panel of human judges, and all results are reported with bootstrap confidence intervals and stochastic replicates. **Results**: Our experiments show that the VLM qwen2.5vl:7b achieves the highest overall performance in food identification ($F_{1}=0.68$), outperforming other open-source models in terms of precision and $F_{1}$ score. Under the human-validated semantic matcher, it also outperforms the more recent successors of the same model families, which nevertheless improve portion estimation and, under a threshold-free LLM judge less sensitive to verbose descriptions, match or exceed it in recognition. For comparison, we evaluate five proprietary models (four OpenAI generations and one Anthropic model) as a benchmark and find that they tend to outperform open-source VLMs in both food recognition (best $F_{1}=0.77$, GPT-4o) and portion size estimation error; notably, however, the most recent proprietary generations do not improve on their predecessors for this task under our evaluation protocol. **Conclusions**: These findings highlight the potential and current limitations of general-purpose VLMs in fine-grained food understanding tasks, with implications for the design of scalable, vision-based dietary assessment systems in digital health.

## 1. Introduction

Automated food recognition from images has emerged as a valuable tool for health monitoring, dietary assessment, and lifestyle tracking [1,2,3,4,5,6,7]. As diet-related chronic diseases such as obesity, diabetes, and cardiovascular conditions continue to rise globally, there is an increasing need for accurate, continuous monitoring of dietary intake and physical activity [8,9]. Traditionally, dietary assessment has relied on self-reporting methods, such as food diaries [10], 24-hour recalls [11], and food frequency questionnaires [12]. However, these approaches are often burdensome, time-consuming, and susceptible to various sources of errors, particularly the underreporting of caloric intake [13]. Their accuracy is further undermined by limited user engagement and the inherent difficulty of estimating portion sizes. Even with visual portion-size aids, mean estimation errors approach 20% for some food types, such as liquids [14]. This highlights the potential of automated food recognition systems to provide more objective, scalable, and user-friendly alternatives to manual dietary reporting. However, automatically identifying food items and estimating portion sizes from photographs remains a challenging problem due to the high visual variability in meals, which might be caused by overlapping ingredients, diverse presentation styles, recipe differences, and inconsistent lighting conditions.

Beyond traditional self-reporting methods, a substantial body of work has explored the application of machine learning techniques, particularly Convolutional Neural Networks (CNNs), for automated food image analysis. Early approaches focused on handcrafted visual features combined with classical classifiers, while later methods leverage deep CNN architectures to learn hierarchical representations directly from raw images [15,16]. CNN-based models have been successfully applied to food classification, ingredient recognition, and food instance segmentation [2,17,18,19,20,21,22]. Despite notable progress, CNN-based systems are typically limited to closed-set classification and require task-specific architectures, extensive labeled data, and careful feature engineering. Moreover, they lack the ability to incorporate contextual and semantic knowledge about food composition, preparation styles, or serving conventions, which constrains their flexibility in real-world, unconstrained settings.

Recent advances in Vision–Language Models (VLMs) [23,24], which integrate the visual understanding capabilities of computer vision with the contextual reasoning of Large Language Models (LLMs), have shown strong performance across a range of image understanding tasks [25,26,27]. While commercial models such as ChatGPT (GPT-4 and GPT-4V) have shown promising capabilities in this domain [28,29,30], and some studies have begun to apply VLMs, including open-source ones, to food identification [31] and vision–language approaches to nutrient estimation [32], comprehensive and systematic benchmarking of these models in this domain remains limited. Recent large-scale evaluations have compared VLMs on aggregate nutrient estimation using public databases such as Nutrition5k [33] and [34,35]; however, item-level benchmarks in which each individual food item is paired with a portion weight measured on a scale, and in which the automated scoring procedure is itself validated against human judgment, are still lacking. Open-source models offer key advantages over commercial alternatives, including the possibility of fine-tuning their parameters for domain-specific applications [36], and the possibility to perform inference locally (for example, on portable devices such as smartphones), without relying on external APIs. This not only reduces dependency on token-based pricing model, which can be costly for input images, but also offers benefits in terms of latency, offline functionality, and user privacy.

A remaining critical frontier is portion size and volume estimation, which is essential for determining total nutrient content in the absence of labels [37]. Current methodologies range from 3D reconstruction and shape templates to the use of reference objects to provide a metric scale [38,39]. While semantic and instance segmentation allow models to localize and count individual food items, predicting the height of amorphous foods from a single 2D image remains complex [40]. Emerging techniques leveraging depth-sensing cameras or CNN-based depth estimation from single RGB images offer a promising path for converting 2D images into voxel grids for precise volume calculation [18].

In this study, we evaluate several state-of-the-art open-source VLMs on the task of recognizing food items from real-world images. Using a curated dataset with ground truth annotations that we collected specifically for this task, we assess each model’s ability to identify food items and estimate corresponding portions. Our goal is to benchmark the current landscape of open VLMs for food-related tasks and provide insight into their strengths and limitations in this domain. The benchmark is built on meals photographed in a university canteen under real-world conditions, a setting that allows every food item to be weighed and all models to be compared on exactly the same images; extending the evaluation to other cuisines and to free-living settings is a natural next step.

This work is structured as follows: In Section 2.1, we present our dataset, combining food items and portion sizes. In Section 2.2, we outline the methods used in this work to assess the performance of the different VLMs, in terms of both food recognition and portion size estimation, on the dataset. Section 3 presents our results, which are then discussed in Section 4 where we also present future works and possible improvements. Finally, in Section 5, we summarize our main results and provide our conclusions.

## 2. Materials and Methods

### 2.1. Data

To evaluate the performance of VLMs on food recognition, we collected and curated a dataset of real-world food images representing a diverse set of meals and presentation styles. A total of 200 high-resolution food images were taken by members of the research team during their regular meals; the resulting set covers a range of food categories, portion sizes, and visual complexity. Some of the images contained several food items (for example, pasta, meat, a side-dish, bread, fruit and a glass of water), while others only a single item, for a total of 870 individual food items (between 1 and 7 items per image, 4.35 on average), corresponding to 314 unique food descriptions. The images were taken under different conditions of lighting and framing. While we tried to ensure a range of cuisines and common meal types, the focus was on dishes from the Italian cuisine. To reduce resolution, each image was resized so that its longest side did not exceed 748 pixels, while maintaining the original aspect ratio.

We labeled food items visible in each image, specifying both the item name (e.g., “grilled chicken”, “steamed broccoli”) and a corresponding portion size in grams. Crucially, all portion sizes were measured using a kitchen scale during data collection, allowing for precise ground truth mass values (in grams). This enabled objective and quantitative comparison of model-estimated portion sizes. Figure 1 shows the histogram of portion sizes, expressed in grams, for the 870 individual food items in the dataset. The mean portion size is ∼152 g, and the median is 129 g. The distribution is positively skewed, with a maximum of 770 g and a minimum value of 10 g.

The annotations were then exported to a structured file, containing image filenames, food item names, and portion sizes. An example of an image and the corresponding label is shown in Figure 2. When labeling the images, we made sure not to be too specific in the description, writing only what could be reasonably discerned by a human viewing the picture. All the food items were weighed when cooked (or, more generally, when ready to be eaten); therefore, the portion sizes do not represent the amount of raw ingredients (e.g., pasta or rice portions). We included both food items and drinks, if present in the image.

This labeled dataset serves as the ground truth for evaluating model predictions. The relatively small size of the dataset allows for detailed qualitative analysis while still enabling quantitative benchmarking. Item names and portion sizes were recorded at the moment of collection by the personnel who gathered the data (experienced researchers, including a nutritionist), and all annotations were subsequently checked manually by the authors to ensure consistency and accuracy across the dataset. Each food item was weighed individually with a kitchen scale, subtracting the tare of the empty plate. Most of the photographed meals (166 images) were the regular dishes served at a university canteen: as the images were contributed by six participants photographing their own meals, the dataset spans a naturally varied menu rather than a curated selection. The exception is a subset of 34 images, contributed by one participant, showing home-prepared lunches, typically consisting of a single dish. Images were collected between 2025 and 2026 using personal smartphones of different makes and models, with no imposed acquisition protocol (photographs were taken freely, mostly from a top-down perspective), so that the dataset reflected the heterogeneous acquisition conditions of real-world dietary logging in terms of device, framing, and lighting.

Table A1 (Appendix A) details the composition of the dataset. Items were grouped into broad food categories by keyword rules applied to their descriptions: the most frequent are bread (160 items); pasta, rice and grains (116); vegetables (97); meat and other protein dishes (93); and fruit (86), while beverages (59 items, almost exclusively water) have the largest median weight (330 g). As expected for a canteen menu, food descriptions recur across photographs: 207 of the 314 unique descriptions occur only once, whereas items whose description occurs at least five times account for 55% of all items (the most frequent being bread, water, and fruit yogurt). The six participants contributed between 8 and 60 images each. Since items photographed together share lighting, perspective, device, and meal context, the 870 items are not independent observations: the image is therefore the unit of statistical analysis throughout (Section 2.2.3), so that the reported uncertainties reflect the number of images rather than the number of items.

### 2.2. Methods

We evaluated several state-of-the-art open-source VLMs on the task of identifying food items and estimating portion sizes from real-world food images. The evaluation consisted of both classification and regression components, reflecting the dual goals of recognizing food types and quantifying their corresponding portion size. A selection of open-source VLMs was used in this study. Table 1 summarizes the models and their main characteristics, including number of parameters and model size. These models combine a vision encoder with a large language model decoder and are capable of reasoning over images.

BakLLaVA is a 7B parameter model designed for efficient multimodal instruction-following and reasoning [41]. Gemma 3 (by Google DeepMind) is released in sizes up to 27B and is designed for efficient deployment with strong multilingual and visual reasoning [42]. Granite Vision 3.2 (IBM) is a compact 2B model tailored to document and chart understanding, offering strong reasoning relative to its size [44]. Llama 3.2-Vision (Meta) extends the Llama family with vision, offering balanced reasoning and perception in 11B and larger variants [45,46]. LLaVA (University of Wisconsin–Madison, Microsoft, and Columbia) combines a CLIP vision encoder with Vicuna, achieving strong instruction-following and conversational multimodal abilities [47]. LLaVA-Llama 3 integrates LLaVA’s tuning with Llama 3 for improved multimodal reasoning in an 8B variant [48]. LLaVA-Phi3 is a 3.8B parameter variant designed to enhance visual reasoning and instruction adherence [49]. MiniCPM-V 2.6 (based on Qwen-2) emphasizes efficiency and on-device inference while retaining competitive accuracy [50,51]. Ministral-3 is available in 3B, 8B, and 14B variants, designed to balance performance and efficiency across multiple scales [54]. Mistral Small 3.1 (Mistral AI) is a 24B model designed to maximize accuracy and reasoning ability, though it requires significantly more compute [55]. moondream 2 is a 1.8B ultra-lightweight model optimized for speed and low resource use, at some cost to accuracy [56]. Qwen2.5-VL (Alibaba Cloud) is available from 3B to 72B parameters and performs well on visual grounding, document understanding, and structured reasoning tasks [57]. Finally, Qwen3-VL is available in 2B, 4B, and 8B variants, offering improved multimodal reasoning and efficiency [58]. To capture the most recent developments in the field, we additionally include the successors of several of these families: Gemma 4 (Google DeepMind), released in edge-optimized (E4B) and 12B variants [43]; MiniCPM-V 4.5 (8B) and the pocket-sized MiniCPM-V 4.6 (1.3B), optimized for on-device inference [52,53]; and Qwen3.5 (4B and 9B), a natively multimodal hybrid-reasoning family [59]. For Qwen3.5, the reasoning mode was disabled during inference, as the extended chains of thought it produces exhaust the context window before an answer is emitted; with reasoning disabled, the models return well-formed outputs.

All models were accessed locally via the Ollama (https://ollama.com/; versions 0.9.6 and 0.33.2; Ollama Inc., Palo Alto, CA, USA) interface to ensure reproducibility and consistent runtime environments. To assess the effect of output variability, we evaluate each VLM at multiple values of the temperature parameter, *T*, which controls the randomness of the model’s output distribution. Lower values of *T* produce more deterministic and conservative responses, while higher values increase diversity and creativity at the cost of consistency. We experimented with the following values of *T*: 0,0.2,0.4,0.6,0.8,1. For the summary comparisons, each model is represented by the temperature that maximizes its $F_{1}$ score at the primary threshold, selected with the same criterion for every model; since this choice is made on the evaluation data itself, the resulting scores are slightly optimistic, an effect we quantify in Section 3. In addition, to characterize the run-to-run variability in the stochastic outputs, we repeated the full 200-image sweep three times (with independent seeds, at T=0.6) for three of the best-performing open-source models (Qwen2.5-VL:7B, Llama 3.2-Vision, and MiniCPM-V 2.6), and verified output determinism at T=0 on a subset of images. Inference seeds, model digests, and software versions were recorded for all runs to ensure reproducibility.

Each image was paired with a standardized prompt designed to elicit detailed food recognition responses, as shown in Appendix B. This prompt encouraged the models to output both food item names and quantity estimates. Model outputs were parsed by the VLM into structured JSON-like formats containing item names and estimated portion sizes. In the cases where the VLM was able to identify food items but failed to produce a valid JSON file, the output was passed to a second LLM, Gemma3:4b, which converted the semi-structured output of the first VLM into a valid JSON file (Some VLMs consistently fail to produce valid JSON, even when explicitly prompted. Across all evaluations, 18.6% of the outputs required this conversion (and 4.5% were empty), with rates ranging from 0% for most models to 100% for models that never return valid JSON (per-model rates in Appendix C). For some models the failures are minor formatting errors, whereas for others the response is a free-text description of the meal, which the repair step converts into the required format; the effect of this step on the results is assessed in Section 3. On the subset of repaired outputs that could be verified deterministically, the repair step preserved 97% of the food item names.). Empty model responses were recorded as empty predictions and were never passed to the repair model, so that no food item could be introduced by the post-processing chain.

Model performance was evaluated along two primary dimensions: food identification performance and portion size estimation. We will now discuss in details the procedure used to assess the performance of each VLM.

#### 2.2.1. Classification: Food Recognition

For the food recognition task, we evaluated how accurately each model could identify the food items present in each image. To this end, we constructed a confusion matrix using the following procedure. Let $L_{\mathrm{GT}}^{i}$ denote the list of ground-truth food item labels for image *i*. $L_{\mathrm{GT}}^{i}$ contains a varying number *N* of items $F_{j}^{i}$, with j=1,…,N. Similarly, let $L_{P}^{i}$ denote the list of *M* predicted food items $f_{k}^{i}$, with k=1,…,M, for the same image *i*. In an ideal case, the matching algorithm would yield M=N, with each predicted item $f_{k}^{i}$ corresponding to exactly one ground-truth item $F_{j}^{i}$. In practice, for every $f_{k}^{i}$ in $L_{P}^{i}$ we determine whether there exists any $F_{j}^{i}$ in $L_{\mathrm{GT}}^{i}$ that constitutes a valid match (we refer to the discussion below for details on the matching procedure). If so, the predicted item is counted as a true positive. Predicted items $f_{k}^{i}$ that do not match any $F_{j}^{i}$ are considered false positives, while ground-truth items $F_{j}^{i}$ not matched by any $f_{k}^{i}$ are considered false negatives. True negatives are not defined in this context.

We then build the confusion matrix by aggregating all correct and incorrect predictions across images, and compute precision, recall, and $F_{1}$ score to evaluate the performance of each VLM.

We identify a match using a Cross Encoder model that computes a similarity score $\eta_{kj}^{i}$ for each pair of texts formed by a predicted food item $f_{k}^{i}$ and each ground-truth label $F_{j}^{i}$ in $L_{\mathrm{GT}}^{i}$. The Cross Encoder is based on a DistilRoBERTa model [60], fine-tuned on the Sentence-Transformers benchmark dataset. Prior to comparison, all food item strings are normalized by removing leading and trailing whitespace and converting all characters to lowercase. A match is considered valid if the similarity score $\eta_{kj}^{i}$ exceeds a given threshold ε∈[0,1]. A threshold of ε=1 enforces exact textual matches, which are uncommon in real-world examples due to variations in naming. Conversely, a value of ε=0 corresponds to accepting completely dissimilar texts as matches. Since the selection of an appropriate threshold is inherently subjective, in the following we report evaluation metrics as a function of ε in order to provide a comprehensive analysis of model performance across varying levels of similarity. Since the $F_{1}$ score of every model decreases monotonically with ε, the threshold cannot be chosen by maximizing performance; it must be anchored externally, which we do through the human validation described in Section 2.2.2. In this work, we explore values of ε in the range 0.5,0.6,0.7,0.8,0.9. We do not consider lower values of ε, as they may lead to a large number of spurious or incorrect matches. When multiple predicted items $f_{k}^{i}$ are matched to the same ground-truth item $F_{j}^{i}$ for image *i*, we retain the prediction with the highest similarity score $\eta_{kj}^{i}$, resulting in a greedy one-to-one assignment in which pairs are accepted in order of decreasing similarity. We verified that replacing this greedy scheme with the globally optimal one-to-one assignment (Hungarian algorithm [61]) changes the $F_{1}$ score by at most 0.006, indicating that the results are insensitive to the choice of matching algorithm.

To illustrate the impact of the similarity score threshold ε on the definition of a correct match, we consider the following examples: With ε=0.5, the pair “pasta with cheese and vegetables” and “baked pasta with tomato sauce and parmesan” is regarded as a correct match (η=0.52), as is the pair “meat” and “vegetable meatballs with radicchio” (η=0.51). By contrast, the pair “cooked diced potatoes” and “baked potatoes” achieves η=0.65, while “cooked spinach” and “boiled spinach” corresponds to η=0.84. Lower thresholds may accept semantically different items as correct, potentially inflating performance. Conversely, higher thresholds enforce stricter semantic similarity, ensuring closer alignment between predicted and ground-truth items, but at the risk of penalizing plausible but not exact matches.

#### 2.2.2. Validation of the Semantic Matcher

Since all recognition metrics depend on the automated semantic matcher, we validated it directly against human judgment. A stratified sample of 300 (prediction, ground-truth) pairs, drawn from the pairs actually encountered during evaluation and covering the full range of similarity scores, was independently annotated by three human judges (the first author and two additional raters not involved in the model evaluation pipeline), who classified each pair as referring to the same food item, to partially overlapping items, or to different items, blind to the similarity scores. Inter-judge agreement was substantial (Fleiss κ=0.60; pairwise Cohen κ=0.54–0.67), with unanimous agreement on 65% of the pairs and complete disagreement on only 2%, indicating that the matching task is well defined. Against the majority vote of the panel, the Cross Encoder score discriminates matches with an area under the ROC curve of 0.88, and the threshold maximizing agreement with the human panel (Youden’s criterion) is ε=0.55 when only “same item” judgments count as matches, and ε=0.52 when partial overlaps are also accepted. These values support the range of ε adopted in this work. The bootstrap 95% confidence intervals of these optimal thresholds are [0.48,0.60] and [0.43,0.55], respectively. We therefore adopt ε=0.5 as the primary operating threshold, being the value of our predefined grid that lies within both intervals: all summary results (Table 2 and the analyses based on it) refer to this threshold, while the other values of ε are reported as sensitivity analyses. The validation pairs were drawn at random (stratified by similarity score) from all the pairs scored during the evaluation; they were used only to select the primary threshold among the predefined grid values and to assess the matcher, and no other component of the pipeline was tuned on them.

A high similarity score does not guarantee that the predicted food is the same as the ground-truth item. We therefore also characterize the matches accepted at the primary threshold: using the majority labels of the validation sample, we estimate for each model the share of accepted matches that correspond to the same food (distinguishing exact textual matches from acceptable naming variations), to partially overlapping foods, or to different foods, by weighting the label distribution observed in each similarity-score interval by the number of matches of the model in that interval. The same estimates yield a strict $F_{1}$ score, in which only matches corresponding to the same food count as true positives.

We further assessed whether the model rankings depend on the specific evaluator. Recomputing all metrics with a second, independently trained Cross Encoder (based on RoBERTa-large [62] instead of DistilRoBERTa, fine-tuned on the same semantic-similarity benchmark) yields rank correlations of ρ=0.94–0.98 (Spearman) with the primary evaluator for ε≤0.8. As a threshold-free alternative, we employed a large language model (GPT-5.2) as a binary judge of food-item equivalence over all 45,993 unique similarity–ambiguous pairs produced by the full model set (plus the 115 new pairs produced by the prompt experiment described in Section 4); the judge only receives pairs of item names, blind to which model produced each prediction (we note that GPT-5.2 is itself among the evaluated models, which is why its per-pair agreement with the human panel, reported below, is the relevant validation): the resulting model ranking correlates with the Cross Encoder ranking at ρ=0.96 (ε=0.5), and the judge agrees with the human majority on the ambiguous pairs (78%, κ=0.55) approximately as well as the human judges agree with one another (75–80%, κ=0.52–0.61). Together, these checks indicate that the reported rankings reflect genuine differences in food recognition ability rather than artifacts of the scoring procedure.

#### 2.2.3. Statistical Analysis

Food items belonging to the same image are not statistically independent. All confidence intervals are therefore computed with a nonparametric bootstrap over images (2000 resamples of the 200 images, with replacement), and comparisons between models use paired bootstrap differences based on the same image resamples. Since true negatives are undefined in this open-vocabulary setting, we avoid the term accuracy and report precision, recall, and $F_{1}$. Run-to-run variability in the stochastic outputs is quantified through the replicate runs described above. Finally, to assess whether the results depend on who acquired the images (and hence on device, framing, and meal type), we repeated the analysis leaving out the images of one participant at a time.

#### 2.2.4. Regression: Portion Size Estimation

For portion size estimation, we conduct a regression-based evaluation. Since all ground truth portion sizes were measured using a digital kitchen scale and recorded in grams, we were able to perform a quantitative comparison between the model-predicted and actual portion sizes.

To evaluate numerical accuracy, we computed the Mean Absolute Error (MAE) and the Median Absolute Error (MedAE) between the predicted and actual portion sizes for each correctly identified food item. The Root Mean Squared Error (RMSE) was also calculated as a secondary measure to reflect the models’ sensitivity to large errors. Predicted portions expressed as ranges (e.g., “100–150 g”) were converted to their midpoint; answers containing no numeric value were treated as missing estimates. These regression metrics provided insight into how well each model could not only detect the presence of food items but also estimate their quantities in a manner consistent with real-world measurements.

One limitation of the standard MAE or RMSE calculation is that they consider only the matched food items, regardless of how many ground-truth items were correctly identified. As a result, a VLM that correctly predicts only a small subset of the items may achieve a deceptively low MAE and RMSE, potentially outperforming another model with higher recognition performance. To address this issue, we introduce a Normalized Mean Absolute Error (NMAE) metric that accounts for both matched predictions and missed detections by penalizing unmatched food items:

(1) $$\mathrm{NMAE}\equiv \frac{\sum_{p=1}^{P}\left|w_{\mathrm{pred},p}-w_{\mathrm{GT},p}\right|+Qw_{0}}{N_{\mathrm{tot}}}\;,$$

where $N_{\mathrm{tot}}$ is the total number of food items in the dataset, $w_{\mathrm{pred},p}$ is the predicted weight (in grams) for the matched food item *p*, $w_{\mathrm{GT},p}$ is the corresponding ground-truth weight, *P* is the number of matched food items with an associated portion estimate, $Q=N_{\mathrm{tot}}-P$ is the number of ground-truth items without a matched numerical estimate (i.e., the missed items plus the recognized items for which no numerical portion was returned), and $w_{0}=129$ g is a fixed penalty assigned to each of these items. The value of $w_{0}$ was chosen to match the median value of the portion size distribution of the labeled dataset, as shown in Figure 1. We note that *P* and *Q* do not necessarily correspond to the numbers of true positives and false negatives defined in Section 2.2.1. For instance, if the VLM correctly predicts the food item label but fails to estimate the portion size, then the item counts as a true positive for the determination of the $F_{1}$ score, but it is penalized according to the weight $w_{0}$ in Equation (1).

The NMAE is well suited for identifying the best-performing models in terms of both portion size estimation accuracy and coverage of ground-truth items. Moreover, it retains the same units as the standard MAE (grams in this case); note, however, that it is not a mean absolute deviation over the estimated items, but an average over all ground-truth items in which each item lacking a matched estimate contributes the fixed penalty $w_{0}$. As a complementary variant, we also report a coverage-penalized MAE (cpMAE) in which each item lacking a matched estimate is penalized by its actual ground-truth weight rather than by the fixed value $w_{0}$:

(2) $$\mathrm{cpMAE}\equiv \frac{\sum_{p=1}^{P}\left|w_{\mathrm{pred},p}-w_{\mathrm{GT},p}\right|+\sum_{q=1}^{Q}w_{\mathrm{GT},q}}{N_{\mathrm{tot}}}\;,$$

where the second sum runs over the same *Q* ground-truth items (missed items and recognized items without a numerical estimate) that are penalized in Equation (1); under this metric, a model that predicts nothing scores the mean portion weight of the dataset (152 g). Neither NMAE nor cpMAE penalizes hallucinated items, which are accounted for only by the precision of the recognition task. We therefore also report two metrics that penalize both omissions and false-positive detections. The first, cpMAE+FP, adds to the numerator of the cpMAE the predicted weight of every false-positive item:

(3) $$\mathrm{cpMAE}+\mathrm{FP}\equiv \frac{\sum_{p=1}^{P}\left|w_{\mathrm{pred},p}-w_{\mathrm{GT},p}\right|+\sum_{q=1}^{Q}w_{\mathrm{GT},q}+\sum_{f=1}^{F}w_{\mathrm{pred},f}}{N_{\mathrm{tot}}}\;,$$

where *F* is the number of false-positive items with a numerical estimate. The second is the meal-level absolute error, i.e., the absolute difference between the total weight predicted for an image (summing all predicted items, matched or not) and the total ground-truth weight of the meal, averaged over the $N_{\mathrm{img}}=200$ images:

(4) $$\mathrm{Meal}\;\mathrm{AE}\equiv \frac{1}{N_{\mathrm{img}}}\sum_{i=1}^{N_{\mathrm{img}}}\left|\sum_{k\in L_{P}^{i}}w_{\mathrm{pred},k}^{i}-\sum_{j\in L_{\mathrm{GT}}^{i}}w_{\mathrm{GT},j}^{i}\right|\;,$$

where the first sum runs over all the predicted items of image *i* that carry a numerical estimate, whether matched or not, and the second over all its ground-truth items (lists $L_{P}^{i}$ and $L_{\mathrm{GT}}^{i}$ of Section 2.2.1). The meal-level error corresponds to the quantity that matters when logging the overall intake of a meal, and is insensitive to how the predicted items are matched to the ground truth.

In summary, every prediction outcome is accounted for as follows: a recognized item with a numerical estimate contributes its absolute error to the item-level metrics (MAE, MedAE, NMAE, cpMAE, and cpMAE+FP) and its predicted weight to the meal total of Equation (4); a recognized item without a numerical estimate and a missed item contribute $w_{0}$ to the NMAE and their true weight to the cpMAE and cpMAE+FP (and, as every ground-truth item, to the ground-truth meal total); a false-positive item does not affect MAE, MedAE, NMAE, and cpMAE, but lowers the precision and, if it carries a numerical estimate, contributes its predicted weight to cpMAE+FP (Equation (3)) and to the predicted meal total (Equation (4)). Finally, an incorrect match (a prediction accepted by the Cross Encoder although it describes a different food) is scored as a recognized item, with its error computed against the weight of the matched ground-truth item; the prevalence of such cases is bounded by the matcher validation of Section 2.2.2 and quantified in Appendix C. The full accounting of outcomes for every model, together with the sensitivity of the NMAE to the value of $w_{0}$, is reported in Appendix C (Table A2).

To contextualize the portion errors, we compare all models against three reference predictors: (i) a null predictor that returns no estimates; (ii) a global-median predictor that, assuming perfect recognition, assigns the dataset median (129 g) to every item; and (iii) a typical-serving predictor that, again assuming perfect recognition, assigns to each item the median weight of the same food computed on the remaining images. On the common cpMAE scale (which, for the latter two predictors, coincides with the plain MAE since no item is missed), the three predictors score 152, 61, and 35 g, respectively; the complete set of metrics is reported in Table 2. The typical-serving predictor quantifies how much of the portion-estimation task can be solved from food identity alone, without any visual size cue: models that do not outperform it on matched items obtain no measurable advantage from visual size cues over typical serving sizes. Unlike the evaluated models, which must first recognize each item from the image, this predictor is given the identity of every food a priori: comparing the two therefore helps to separate the contribution of visual size cues from that of dietary priors on typical serving sizes, a distinction that is relevant for the design of automated nutrition monitoring systems. Note that this predictor is a diagnostic upper bound rather than a deployable baseline: it assumes perfect recognition and draws on the portion distribution of the dataset itself, falling back to the global median for the 24% of items whose food appears in a single image.

Because the evaluated models and the typical-serving predictor are scored on different sets of items (the former only on the items they recognize and quantify), we also compare them on the same items: for every model, the typical-serving estimate is computed for exactly the matched items for which the model returned a numerical portion. On these items we further test whether the model estimates add information to food identity, in two ways: (i) by fitting, with leave-one-image-out cross-validation, a linear combination of the typical-serving estimate and the model estimate, and comparing its MAE with that of the typical-serving estimate alone; and (ii) by computing, for the foods that occur at least five times among the matched items, the correlation between model estimates and true weights after subtracting the mean of each food, i.e., whether the model tracks how large a given portion is relative to other portions of the same food. Since an absolute error has different implications for small and large portions, we also report the median absolute percentage error, $\mathrm{MedAPE}=\mathrm{median}\left(|w_{\mathrm{pred}}-w_{\mathrm{GT}}|/w_{\mathrm{GT}}\right)$.

#### 2.2.5. Proprietary Baselines

We compare the performance of the VLMs to five proprietary models, using them as reference baselines. In particular, we evaluate GPT-4o, GPT-4.1, GPT-5.2, GPT-5.5 (OpenAI, San Francisco, CA, USA), and Claude Sonnet 5 (Anthropic, San Francisco, CA, USA). GPT-4o is a multimodal foundation model capable of processing text and visual inputs, historically serving as a flagship multimodal offering. GPT-4.1, introduced in 2025, provides improved reasoning, instruction following, and long-context comprehension compared to earlier GPT-4-series models. GPT-5.2 and GPT-5.5 represent successive generations of OpenAI’s flagship models, offering substantial improvements in reasoning and multimodal understanding, while Claude Sonnet 5 is a recent general-purpose multimodal model from a different provider. Using these models as baselines allows us to benchmark open-source systems against a spectrum of commercial options that trade off speed, cost, and accuracy in different ways. We note that the most recent proprietary models (GPT-5.5 and Claude Sonnet 5) no longer expose a temperature parameter; for these, we performed three independent replicate sweeps at the provider default in place of the temperature grid and report means and standard deviations across replicates.

We note that GPT-oss, OpenAI’s open-weight models, currently do not support vision-based tasks and were therefore not included in this evaluation.

## 3. Results

Figure 3 and Figure 4 present the result of the model comparison based on the dataset introduced and described in Section 2.1. We plot the $F_{1}$ score as a function of both the model temperature *T* and of Cross Encoder threshold ε, for all open-source VLMs listed in Table 1; the corresponding results for the five proprietary baselines of Section 2.2.5 are shown in Figure 5. In general, we observe a trend where performance improves at lower values of both *T* and ε. A lower threshold ε increases the number of matches by relaxing the similarity criterion between predicted and ground-truth labels, so it naturally improves the assigned number of correct matches. On the other hand, a plausible explanation for the effect of temperature is that lower temperature values reduce the variability in model output, resulting in more focused and less speculative predictions, which also results in an increased number of matches.

Among the open-source models, Qwen2.5-VL:7B achieves the best overall performance, with the highest precision and $F_{1}$ score among open-source models across different settings (see Table 2 for a summary of all metrics at each model’s best configuration). Its $F_{1}$ score ranges from a minimum of 0.12 (at T=1.0, ε=0.9) to a maximum of $F_{1}=0.68$ (at T=0, ε=0.5), with a bootstrap 95% confidence interval of [0.66,0.71]. A more balanced configuration (T=0, ε=0.6) yields an $F_{1}$ score of 0.57. Other VLMs that attain $F_{1}$ scores of at least 0.5 include Qwen3.5 (9B and 4B), MiniCPM-V 4.5, Llama 3.2-Vision, MiniCPM-V 2.6, Mistral Small 3.1, Qwen2.5-VL (3B), Gemma 3 (12B), Gemma 4 (12B), and Qwen3-VL (4B and 8B). The superiority of Qwen2.5-VL:7B among open-source models is statistically robust: in paired bootstrap comparisons at ε=0.5, it outperforms every other open model in essentially all image resamples (P≥0.98), including its own more recent successors (under the threshold-free LLM judge of Section 2.2.2, however, Qwen3.5:9B and MiniCPM-V 4.5 reach or exceed it; see below). Across the three stochastic replicates at T=0.6, its $F_{1}$ is 0.644±0.016, confirming that run-to-run variability (also visible in the replicate panels of Figure 5) is an order of magnitude smaller than the differences between models. The ranking is also robust to the composition of the dataset: leaving out the images of any one of the six participants yields rankings with Spearman correlations of 0.99–1.00 with the full ranking, with Qwen2.5VL:7B always the best open-source model and GPT-4o the best model overall. Absolute performance, however, varies across subsets: on the home-prepared single-dish meals, all models score lower (e.g., $F_{1}=0.66$ for GPT-4o and 0.53 for Qwen2.5VL:7B, against 0.75–0.84 and 0.65–0.76 on the images of the other participants), mainly through a lower precision (e.g., 0.60 versus 0.77 for GPT-4o), as the models tend to describe a single mixed dish as a list of separate ingredients (e.g., “rigatoni pasta”, “black olives”, and “diced tomatoes” for “pasta with tuna, olives, tomatoes and mozzarella”).

At the primary threshold, an estimated 70–81% of the matches accepted for each model correspond to the same food according to the human panel (exact textual matches account for 1–38% of the matches, the remainder being naming variations, such as “cooked spinach” for “boiled spinach”), 10–18% to partially overlapping foods (e.g., “boiled pasta” for “pasta with pesto”), and 8–14% to different foods (e.g., “salad with greens and vegetables” for “baked pasta with vegetables”). Counting only same-food matches as successes lowers the $F_{1}$ scores (e.g., from 0.77 to 0.59 for GPT-4o and from 0.68 to 0.56 for Qwen2.5VL:7B), but leaves the ranking essentially unchanged (Spearman ρ=0.99), with Qwen2.5VL:7B still the best open-source model; the ranking is equally stable at ε=0.55 and 0.6 (ρ=0.99 and 0.96). Conversely, the matcher misses some correct identifications, typically when a verbose prediction describes a common item (e.g., “whole grain or sourdough bread slice” for “bread”, η=0.49, or “small canned yogurt (e.g., tofutti brand)” for “fruit yogurt”, η=0.30), which penalizes the most verbose models. From a nutritional perspective, partially overlapping and incorrect matches are not equivalent to correct identifications, as they may imply a different composition (e.g., a vegetable salad instead of a pasta dish): absolute $F_{1}$ values at the primary threshold should therefore be read as upper bounds of exact food identification, whereas the comparison between models is robust to this distinction.

Figure 6 and Figure 7 show the results in terms of the NMAE, as defined in Equation (1), plotted as a function of *T* and ε. As with the $F_{1}$ score, the NMAE tends to be lowest (better portion estimation performance) for low values of both *T* and ε; the corresponding results for the proprietary baselines are shown in Figure 8. Some VLMs, such as moondream:1.8b, bakllava:7b and granite3.2-vision:2b, provide almost no usable portion estimates even when explicitly instructed to do so in the prompt (see Appendix B): their NMAE remains close to the 129-g value that corresponds to returning no portion estimate at all. Among the open-source models that do provide consistent outputs, Qwen2.5vl:7b achieves the lowest NMAE of its generation, with a value of 80 g at T=0 and ε=0.5 (its minimum, 79 g, is attained at T=0.2); values below 100 g are also attained by Mistral Small 3.1 (86 g), Gemma 4 (12B, 88 g), Gemma 3 (12B, 91 g), and the Ministral-3 family (92–98 g). The 2026 successors improve markedly on this axis: Qwen3.5:9B reaches an NMAE of 70 g and MiniCPM-V 4.5 of 73 g, the best open-source values observed in this work under this metric (metrics that also penalize hallucinated items are discussed with Table 2 below).

The comparison between successive generations of the same model families reveals an asymmetric pattern. On item-level recognition, the most recent model generations do not improve on their predecessors in this benchmark: the best $F_{1}$ among open models still belongs to Qwen2.5-VL:7B (released in 2025), whose successors Qwen3-VL ($F_{1}=0.50$) and Qwen3.5:9B ($F_{1}=0.65$) do not match it, while Gemma 4 performs on par with Gemma 3 ($F_{1}=0.53$ vs. 0.51 at 12B) and only MiniCPM-V 4.5 clearly improves on its predecessor (0.65 vs. 0.57). This conclusion depends, however, on the evaluator: under the threshold-free LLM judge, which credits correct but verbose descriptions falling below the similarity threshold, the successors improve on their predecessors (Qwen3.5:9B 0.65 vs. Qwen2.5-VL:7B 0.61; MiniCPM-V 4.5 0.64 vs. 0.48; Gemma 4 0.48 vs. 0.40 at 12B), suggesting that part of the gap may reflect a scoring penalty on verbose descriptions; a dedicated prompt experiment, however, shows that the ordering of the two open-source generations also depends on the prompt (Section 4). On portion estimation, by contrast, the newer generations improve consistently, reducing the matched-item MAE by roughly 25% (e.g., from 54 to 41 g between Qwen2.5-VL:7B and Qwen3.5:9B).

Since the models were run under partly different conditions, we checked that these differences do not drive the comparison between generations (Appendix C, Table A3). First, output repair: all models in the upper part of the ranking (the five proprietary baselines, Qwen2.5VL:7B, Qwen3.5, and MiniCPM-V 4.5) almost never required it, and on a common subset of images, treating each repaired output as an empty prediction, or replacing the repair model with a deterministic, tolerant JSON parser alone, leaves their $F_{1}$ scores unchanged (by at most 0.003). The repair step mainly benefits the models that rarely return valid JSON, such as Llama 3.2-Vision, the LLaVA family, and Qwen3-VL, whose $F_{1}$ would otherwise drop close to zero; since it can only raise the scores of these models, it cannot explain why older models outperform their successors. Second, temperature selection: reporting each model at its best temperature slightly favors the models evaluated over the full temperature grid with respect to GPT-5.5 and Claude Sonnet 5, which were run at the provider default. However, the ranking based on the mean $F_{1}$ over temperatures correlates with the best-temperature ranking at ρ=0.97, Qwen2.5VL:7B is the best open-source model at every temperature, and at the default temperature of the OpenAI models (T=1) the proprietary ordering is unchanged (GPT-4o 0.75, GPT-4.1 0.73, Claude Sonnet 5 0.71, GPT-5.5 0.66, GPT-5.2 0.65). We therefore conclude that, under the prompt, inference settings, and evaluation procedure of this benchmark, the newer models did not outperform their predecessors in item-level recognition as measured by the Cross Encoder, whereas under the LLM judge this holds only for the proprietary models; whether this holds under other prompting strategies remains to be established.

We identify Qwen2.5VL:7B as the best open-source model for food recognition, as well as among the best of its generation for portion size estimation. In the left panel of Figure 9, we show the ground-truth portion sizes as a function of the portion sizes predicted by Qwen2.5VL:7B at the configuration depicted in Table 2 (T=0, ε=0.5). The black dashed line indicates the identity line, while the red solid line shows the linear best fit to the data. Although a positive correlation is clearly evident, the model generally tends to under-predict portion sizes, with deviations from the identity line becoming larger for larger portion sizes. We also observe that this VLM tends to predict portion sizes in discrete steps, typically in multiples of 50 g (95% of its estimates).

The tendency to under-predict large portions is not specific to this model. Considering the matched items of eight representative models (the three best open-source models and the five proprietary baselines, based on the configurations depicted in Table 2), the estimates for portions below the median weight of these matched items (126 g) are nearly unbiased, with median signed errors within ±16 g, whereas for the largest quartile of portions (above 187 g), all eight models under-predict, with median errors ranging from −33 g (Qwen3.5:9B) to −94 g (Qwen2.5VL:7B). The under-prediction is strongest for beverages, whose median ratio between predicted and true weight is 0.71 (with 84% of the estimates below the true value): for water, whose true weight has a median of about 330 g, nearly all estimates are 200 or 250 g. Fruit (0.83) and vegetables (0.84) follow, while soups are over-predicted (1.16). The most recent models are unbiased on the median item (ratios of 1.00–1.05 for Qwen3.5:9B, GPT-5.2, GPT-5.5, and Claude Sonnet 5), yet they still under-predict the largest portions.

Table 2 summarizes all evaluation metrics—$F_{1}$ with bootstrap confidence intervals, MAE, MedAE, NMAE and cpMAE—for every model at its best-performing configuration, together with the reference predictors. Comparing the models against the reference predictors puts these errors in perspective. All usable models clearly outperform the null predictor (cpMAE =152 g): the best open-source values are 77–80 g (Qwen3.5:9B and MiniCPM-V 4.5), and the best proprietary values reach 68–70 grams. On the matched items, however, no model outperforms the typical-serving predictor (MAE =35 g): the best matched-item MAE is 35 g for GPT-4o and 41 g for Qwen3.5:9B. In other words, for this dataset, the models’ portion estimates are, at best, comparable in accuracy to assigning each recognized food its typical serving size. This likely reflects in part the standardized portioning of canteen meals, which makes food identity highly informative about weight; nevertheless, it suggests that current VLMs extract little additional size information from the image itself.

The ranking of the models by NMAE is robust to the choice of the penalty: among the 26 models with more than 50 numerical estimates, halving or doubling $w_{0}$ (64.5 or 258 g) yields Spearman rank correlations of 0.93 and 0.995 with the reference ranking, penalizing missed items by their true weight (cpMAE) yields 0.99, and re-scoring as errors the matches that the LLM judge of Section 2.2.2 considers incorrect (9–14% of the matches of the best models) yields 0.99 (Appendix C). Penalizing hallucinated items changes the picture more substantially (ρ=0.67 for cpMAE+FP): GPT-4o remains the best model (96 g), but among open-source models Qwen2.5VL:7B (113 g) overtakes Qwen3.5:9B (131 g), and the most recent proprietary models fall behind their predecessors (127–132 g for GPT-5.2 and GPT-5.5), reflecting the larger number of spurious items they report. At the level of whole meals, the opposite holds: the most recent models attain the lowest meal-level errors (118–125 g for Qwen3.5:9B, GPT-5.2, GPT-5.5, and Claude Sonnet 5, i.e., a median relative error of 15–17% of the meal weight), ahead of GPT-4o (157 g) and Qwen2.5VL:7B (243 g), because the weight of their additional items partly compensates for the under-estimation of the recognized ones. Which model is preferable for portion estimation therefore depends on whether errors are assessed item by item or on the total intake of a meal.

Table 3 compares the models with the typical-serving reference on exactly the same items. The conclusion above holds under this matched comparison: on the items quantified by each model, the typical-serving reference attains an MAE of 33–39 g and a MedAPE of 12–16%, against 35–54 g and 17–29% for the models, and a cross-validated linear combination of the two estimates does not improve significantly on the reference for any model. Nevertheless, the model estimates are not uninformative about the specific portion in the image: within the same food, the estimates of most of the best models correlate positively with the true weight (r=0.13–0.25 for GPT-4o, GPT-4.1, GPT-5.2, Claude Sonnet 5, Qwen3.5, and Gemma 4 12B, with confidence intervals excluding zero), whereas no significant correlation is found for Qwen2.5VL:7B, MiniCPM-V 4.5, Mistral Small 3.1, and GPT-5.5. These results indicate that current VLMs do extract visual information on portion size, but that, in this dataset, the precision of their estimates is not yet sufficient for this information to improve on what food identity alone conveys.

### 3.1. Proprietary Baseline Performance

Figure 5 and Figure 8 show the results for the five proprietary baseline models discussed in Section 2.2.5. The resulting $F_{1}$ score and NMAE do not depend significantly on the chosen value of the temperature (nor, for the most recent models, on the replicate), while, as expected, the $F_{1}$ decreases monotonically with increasing values of ε. The highest value of $F_{1}$ score is attained by GPT-4o, with $F_{1}=0.77$ for ε=0.5 (bootstrap 95% CI [0.75,0.80]), followed by GPT-4.1 ($F_{1}=0.75$), Claude Sonnet 5 (0.70±0.01 across replicates), GPT-5.2 (0.67), and GPT-5.5 (0.66±0.004). For ε=0.6, the values for the OpenAI models are in the range [0.41,0.57].

The lowest NMAE among all models tested in this work is attained by the proprietary baselines: 58 g for GPT-4o, 62 g for Claude Sonnet 5, and 63 g for GPT-4.1 (at ε=0.5). Notably, the ordering of the proprietary models by $F_{1}$ is anti-chronological: each successive OpenAI generation performs slightly worse than its predecessor on item-level recognition, from GPT-4o (0.77) down to GPT-5.5 (0.66), a difference that far exceeds the replicate variability.

In the right panel of Figure 9, we report the performance of GPT-4o in reconstructing the portion size of individual food items. The model is nearly unbiased for small and medium portions, whereas it systematically under-predicts the largest ones, with errors growing to several tens of grams (the distribution of the errors by portion size was quantified above for eight representative models). In comparison, the open-source VLM Qwen2.5VL:7B, shown in the left panel of Figure 9, produces outputs that are more discrete, whereas GPT-4o generates more continuous and smoothly varying predictions.

The declining trend of the newer generations is somewhat unexpected, but can be further analyzed by examining individual inferences. For instance, for a temperature T=0.6, the output of GPT-5.2 for the image in Figure 2 (extracted from the JSON file) is: “pasta shells with beans (likely cannellini) and grated cheese” (280 g), “slice of savory pie/quiche (egg-based) with herbs/cheese” (130 g), “diced sauteed zucchini (courgette) side” (160 g), “bread roll (in plastic bag)” (70 g), “apple (whole)” (170 g), “water (in plastic cup)” (250 g). By comparison, GPT-4.1 produces: “pasta with mushrooms and grated cheese” (180 g), “slice of vegetable quiche” (90 g), “zucchini and potato sauteed” (100 g), “plain bread roll” (50 g), “red apple” (120 g), which, despite the omission of the water, aligns more closely with the ground-truth labels shown in Figure 2. Notably, the output of GPT-5.2 is more detailed and complex, which may negatively impact the food recognition task when using the Cross Encoder model to compute similarity scores above a fixed threshold. The same pattern of increasingly verbose, attribute-rich descriptions is observed for GPT-5.5 and, to a lesser extent, Claude Sonnet 5, whose $F_{1}$ also degrades faster than that of GPT-4o as ε increases. Importantly, the validation of Section 2.2.2 shows that this ordering is not an artifact of the Cross Encoder: the same ranking is recovered by a threshold-free LLM judge whose decisions agree with human judges about as well as humans agree with each other, although with a smaller gap between GPT-4o and GPT-5.5 (0.72 vs. 0.66, against 0.77 vs. 0.66 with the Cross Encoder). Section 4 examines two candidate mechanisms for this pattern, the verbosity of the predicted names and the tendency to list more items than the human annotators (over-itemization), and tests the first with a dedicated prompt experiment, whose outcome points to over-itemization as the main factor.

### 3.2. Calibration of the Weight Estimation

By comparing the predicted portion sizes to the true measured values, we calibrate the output of the different VLMs to correct for systematic biases. Inspired by the trends shown in Figure 9, we adopt a simple linear model to describe the relationship between predicted and ground-truth weights for each VLM:

(5) $$w_{\mathrm{GT}}=b+kw_{\mathrm{pred}}.$$

The parameters *b* and *k* are specific to each model and are determined, together with their covariance matrix, via a least-squares linear fit on the *P* matched food items with an associated portion estimate. To avoid evaluating the correction on the same data used to fit it, the calibrated errors are computed with a leave-one-image-out cross-validation, in which the calibration line applied to the items of each image is fitted on the items of all other images. The effect of the calibration depends on the model (Table 4). It is beneficial for Qwen2.5VL:7B (left panel of Figure 9), whose estimates are systematically too low by a factor that grows with the portion (k=1.30): its median absolute error (MedAE) decreases from 40 to 32 g and its MAE from 54 to 47 g. For GPT-4.1, the calibration reduces the MAE (44 to 40 g) but leaves the MedAE unchanged, and for all other models, it does not reduce the error: for GPT-4o, whose fitted line is close to the identity (k=1.00), the MedAE changes from 19 to 22 g, and for the most recent models (Qwen3.5:9B, MiniCPM-V 4.5, GPT-5.2, GPT-5.5, and Claude Sonnet 5) the MedAE increases by 2–11 g. For these models the raw median error is already low and the residual error is dominated by the under-prediction of the largest portions (Section 3), which a single straight line, fitted by least squares on all items, corrects at the expense of the small and medium portions. Cross-validated and in-sample calibrated errors agree to within 0.3 g, indicating that the fitted lines generalize across images. We stress that MedAE values for a given VLM should be interpreted with care, as deceptively low errors may arise from a small number of matched ingredients (see discussion in Section 2.2.4).

Table 4 reports the fitted values of the parameters *b* and *k* for a representative subset of models (the top open-source performers and the five proprietary baselines), at the configurations of Table 2. We can see how, even if the proprietary models result in lower values of MedAE, the VLM Qwen2.5VL:7B exhibits the lowest value of the intercept *b*, together with the largest slope. A lower intercept indicates a smaller systematic offset in the predicted portion sizes at low weights, suggesting reduced bias for small servings. The most recent models, open-source and proprietary alike, converge instead to a common regime with slopes below unity (k=0.71–0.89) and intercepts of 30–46 grams, which reflect the compression of their estimates toward typical values: small portions are slightly over-predicted and large portions under-predicted. Since their raw median errors are already low (20 g for GPT-5.5 and Claude Sonnet 5), the linear correction does not further reduce the MedAE for these models.

## 4. Discussion

Our results highlight notable differences in performance across open-source VLMs when applied to food recognition tasks. As illustrated by Figure 3, Figure 4 and Figure 5, the performance of the models depends strongly on the threshold value used to identify good matches between the model predictions and the labeled dataset. More stringent matching criteria result in a lower number of matches, and therefore reduce the recognition performance; the human validation of Section 2.2.2, however, anchors the operating range of ε to human judgment and shows that the model rankings are stable across evaluators. Among the evaluated models, Qwen2.5VL:7B achieves the highest open-source $F_{1}$ (0.68), followed by Qwen3.5:9B (0.65) and MiniCPM-V 4.5 (0.65); about ten further configurations reach $F_{1}≳0.5$ for selected parameter settings.

Portion estimation proved more challenging across all models. While most models were able to identify common food items with reasonable accuracy, estimating portion sizes, a more quantitative and spatially sensitive task, resulted in larger errors. Even the top-performing models exhibited noticeable discrepancies from the ground truth, as measured by the MAE, MedAE and NMAE. The comparison with the typical-serving predictor sharpens this point: since no model outperforms a predictor that assigns each recognized food its typical serving size, the portion estimates of current VLMs appear to be driven primarily by learned priors on food identity rather than by visual size cues extracted from the image. The within-food analysis of Table 3 qualifies this conclusion: the best models do respond to the size of the portion in the image, but weakly, so that their failure to outperform the typical-serving reference reflects the limited precision of their visual estimates rather than an absence of visual information. We note that canteen meals, with their standardized portioning, make such priors unusually effective, so this conclusion may be less severe in free-living settings with more variable servings. We hypothesize that this limitation reflects a lack of exposure to portion-estimation tasks during training and the absence of scale or reference objects in many food images, in addition to the inherent ambiguity of inferring physical size from a single two-dimensional view. The structure of the errors offers some indication of their origin, although the following explanations remain hypotheses that our design does not test directly. First, the concentration of the under-prediction in the largest portions is consistent with estimates being compressed toward typical serving sizes, as expected if they rely on learned priors. Second, the under-prediction of beverages, whose estimates cluster at 200–250 g regardless of the actual amount, suggests that models default to the nominal capacity of a standard glass rather than reading the filling level, which is hard to judge from a top-down view of a transparent cup. Third, a single RGB image provides no depth information and, in our photographs, no calibrated reference object, so that the height of piled or deep-dish foods is intrinsically ambiguous; partial occlusion between items on a crowded tray may also contribute, but it was not annotated in our dataset and could not be quantified. By contrast, constraints on output length can be excluded as a cause: portion sizes are requested as a short numeric field, and none of the 1600 responses of the eight models discussed above was truncated (only one required JSON repair). Disentangling the remaining factors would require images with depth data or reference objects, together with occlusion annotations.

A further pattern emerging from our benchmark is that, under our prompt and evaluation protocol, recent model generations, both open-source and proprietary, did not clearly improve item-level food recognition. Among the proprietary baselines the ordering is anti-chronological, with GPT-4o outperforming all of its successors, and among the open families the 2025-generation Qwen2.5-VL:7B remains unmatched under the Cross Encoder, although not under the LLM judge. We considered two mechanisms that could produce this pattern. The first is verbosity: within each family, newer models describe foods with longer, attribute-rich names (on average 3.0 words per item for GPT-4o, 4.0 for GPT-4.1, and 5.2 for GPT-5.2; 2.4 for Qwen2.5-VL:7B and 5.1 for Qwen3.5:9B, against 2.5 for the ground-truth labels), which lowers their textual similarity to concise human labels even when the food is correctly identified; consistently, the gap between generations shrinks under the threshold-free LLM judge. The second is over-itemization: newer models list more items per image (4.5 for GPT-4o versus 5.4 for GPT-5.5, against 4.35 in the ground truth), so that more of their predictions remain unmatched and their precision decreases (from 0.77 to 0.60 between GPT-4o and GPT-5.5), while recall remains stable (0.72–0.78 across the proprietary models); this component persists under the LLM judge (precision 0.71 vs. 0.60). To test the first mechanism directly, we re-ran two older/newer pairs (GPT-4o and GPT-5.5; Qwen2.5-VL:7B and Qwen3.5:9B) at their configurations of Table 2, with a prompt that differed from the original only in requesting the shortest common name of each food (Appendix D, Table A4). The prompt shortened the names substantially (1.3–1.5 words per item, fewer than in the human labels) but did not reduce the number of listed items, and it did not close the gap between the proprietary generations: GPT-5.5 did not benefit from it ($F_{1}=0.65$ with the Cross Encoder, and a slightly lower LLM-judge score than with the original prompt), whereas GPT-4o lost part of its advantage under the Cross Encoder ($F_{1}$ from 0.77 to 0.72) but not under the LLM judge (0.72 in both cases), and remained ahead of GPT-5.5 under both evaluators. For the open-source pair, the two generations were on par under the concise prompt (Cross Encoder $F_{1}=0.66$ for both; LLM judge 0.64 vs. 0.62). These results suggest that the lower recognition scores of the newer proprietary models stem mainly from over-itemization rather than from name length, and that what matters for the matching is consistency with the granularity of the human labels rather than brevity per se; for the open-source families, the ordering between generations depends on the evaluator (the successors match or exceed Qwen2.5-VL:7B under the LLM judge) and on the prompt. Name length is not, moreover, a general determinant of performance: across all models, it is not correlated with the $F_{1}$ score (Spearman ρ=0.22). At the same time, the newer generations do improve portion estimation, which might indicate, as a hypothesis that our data cannot test, that recent training emphasizes quantitative and descriptive detail over concise categorical naming. In practice, our experiment shows that requesting shorter names alone does not remove this penalty, as it also discards information that human labels retain. More promising strategies, which remain to be tested, include separating the food name from its attributes (cooking method, ingredients, container) in the output schema, enforcing the schema through structured-output decoding, normalizing the predicted names to a food-composition vocabulary before matching, and explicitly instructing the models to list each dish once rather than its individual components, which would target over-itemization. For dietary-assessment applications, this indicates that the newest general-purpose flagship model is not necessarily the best choice, and that model selection should be validated on the target task.

To partially mitigate systematic biases in portion estimation, we explored a simple linear calibration of model predictions. While this post hoc correction does not address the underlying perceptual limitations of the models, it can reduce consistent over- or under-estimation trends; in our data, however, it improved the median absolute error only for the model with the largest systematic bias (Qwen2.5VL:7B), whereas for the most recent models, whose errors are concentrated in the largest portions, a global linear correction brought no benefit.

Finally, some models such as qwen3.5:9b and mistral-small3.1:24b showed more consistent performance across both tasks, while others, such as llama3.2-vision:11b, displayed trade-offs, such as excelling in classification but underperforming in portion estimation. This could inform future model selection or fusion strategies depending on the specific application, such as dietary logging, meal tracking, or clinical nutrition analysis.

Many prior works on food recognition with VLMs or related models have framed the task as a fixed multi-class classification problem, where foods in the dataset are organized into predefined categories, subcategories, or cooking styles, and model performance is evaluated within this rigid structure [31], or have evaluated aggregate nutrient estimates at the level of the whole dish [34,35]. In contrast, our approach does not assume any predefined class taxonomy, and evaluates each individual food item against a scale-measured weight. Instead, we directly assess whether a model can match an image to its corresponding ground-truth label, which is unknown to the model a priori. This setup makes the task considerably more challenging: rather than choosing from a closed set of classes, the model must generalize across a large and diverse label space. As a result, the values we report are lower than those typically seen in classification-based benchmarks. Moreover, our setup is more prone to apparent misclassification errors when synonyms or near-equivalent terms are not correctly recognized as a match. For this reason, the comparison to strong proprietary baselines such as OpenAI’s models is especially important, as relative performance provides a more meaningful indicator than the absolute values of metrics like $F_{1}$ score or NMAE.

The generalizability of these results to other food cultures deserves specific consideration. Our dataset reflects Italian canteen meals, which consist of a few separately served and visually distinct items (typically a first course, a main course, a side dish, bread, and fruit) described with relatively standard names. Cuisines in which several components are mixed in a single dish or served family-style, in which sauces and fillings hide the ingredients, or whose dishes have regional names without a standard English equivalent are likely to make both item-level recognition and its automated scoring harder, and the relative performance of the models may also change, depending on how well each cuisine is represented in their training data. Likewise, portion priors learned from Western serving conventions may transfer poorly to cuisines with different serving sizes or shared plates, which would make portion estimation harder than in our canteen setting. These expectations are hypotheses that a multi-cuisine extension of the benchmark, together with the re-validation of the matching threshold discussed below, would allow testing.

Some limitations of this study should be noted. Beyond the single-site, single-cuisine origin of the dataset discussed above, the strong typical-serving prior is itself a property of standardized institutional meals. Performance in free-living dietary assessment, where meals are more varied, portions less standardized, and photographs taken under less controlled conditions, may differ from the figures reported here, as suggested by the lower scores obtained on the home-prepared subset. External validation on an independent dataset was not possible, since, to our knowledge, no public collection pairs item-level descriptions of the visible food items with individually weighed portions: existing resources such as Nutrition5k [33] provide ingredient-level masses of whole dishes rather than per-item weights. Assembling such multi-site, multi-cuisine collections is therefore a priority for future benchmarks. In addition, inference settings could not be fully harmonized across models: the most recent proprietary models do not expose a temperature parameter, the reasoning mode of Qwen3.5 had to be disabled, and some models depend on the repair step to produce structured outputs; although we verified that these differences do not drive the main comparisons, all models were evaluated with a single prompt, and other prompting strategies may change their relative performance. The dataset size (200 images, 870 items) enables detailed item-level analysis and statistically resolved model comparisons, but larger multi-site collections will be needed to characterize generalization. Finally, while the semantic matcher was validated against a human panel, any text-based scoring of open-vocabulary predictions retains a degree of irreducible ambiguity, which we quantify (2% of validation pairs with complete inter-judge disagreement) rather than eliminate. Moreover, since the matcher was validated on English-language descriptions of Italian canteen dishes, in the heterogeneous settings discussed above the score distributions of correct and incorrect matches may shift, and the optimal threshold ε cannot be assumed to transfer. Applications in such settings should re-validate the threshold on a small human-annotated sample of pairs, following the protocol of Section 2.2.2 (whose annotation form and scripts are publicly released), and may benefit from multilingual cross-encoders or from LLM-based judges, which in our validation agreed with the human panel about as well as the human judges agreed with one another.

A particularly promising direction for future work is the systematic fine-tuning of open-source VLMs for nutrition-related tasks. Because these models are relatively small and openly available, they can be adapted to specialized domains without prohibitive computational cost. Fine-tuning could involve (i) supervised adaptation using curated food image–label pairs, (ii) instruction tuning on nutrition-related prompts to align model outputs with dietary contexts, or (iii) contrastive learning with embeddings that map foods to semantic or nutritional descriptors. Domain-specific fine-tuning may also help mitigate issues such as synonym mismatches (e.g., “zucchini” vs. “courgette”) or regional naming variations, leading to more robust label matching. Furthermore, continual fine-tuning with user-generated data could support personalized applications, where the model adapts to individual dietary habits over time.

## 5. Conclusions

In this study, we evaluated twenty-six configurations (seventeen model releases) of state-of-the-art open-source VLMs on two tasks: food item identification and portion size estimation from images of meals collected in a university canteen. The selected VLMs represent relatively small-scale architectures (≤15 GB in size and fewer than 25B parameters), which makes them substantially smaller than proprietary models such as those from OpenAI and Anthropic. Their reduced footprint offers practical advantages: they can be deployed for on-device inference, thereby reducing latency, improving safety and privacy, and enabling use in resource-constrained environments such as mobile health applications. Moreover, being open source, these models are amenable to fine-tuning for specific domains, allowing adaptation to diverse nutritional or dietary contexts. To support this evaluation, we curated a dataset comprising 870 distinct food items, each paired with human-annotated labels and human-measured portion weights.

Within the scope of this benchmark, i.e., for canteen meals, a single prompt, and the evaluation pipeline described above, our findings can be summarized as follows:

- The performance of food item identification is strongly influenced by the similarity score threshold used to determine a correct match; a human-validated calibration of the semantic matcher anchors this threshold to human judgment (optimal ε≈0.52–0.55) and shows that the model rankings are overall robust to the choice of evaluator.
- While most models reliably recognize common food items, accurate portion size estimation remains a significant challenge: no model outperforms a predictor that assigns each recognized food its typical serving size, suggesting that current VLMs rely on identity-based priors more than on visual size cues, although the estimates of the best models retain a weak but measurable sensitivity to the actual portion.
- Proprietary models generally outperform open-source VLMs on this benchmark, achieving higher recognition performance and lower errors in portion size estimation; however, within both groups, the most recent generations do not improve item-level recognition under the prompt and evaluation protocol used here, and the best results are obtained by GPT-4o and Qwen2.5VL:7B rather than by their successors, although for the open-source models, the ordering between generations depends on the evaluator and on the prompt, with the successors matching or exceeding Qwen2.5VL:7B under a threshold-free LLM judge.
- Among the twenty-six open-source VLM configurations tested, Qwen2.5VL:7B exhibits the best recognition performance under the human-validated matcher ($F_{1}=0.68$, with P≥0.98 of ranking first among open models in paired bootstrap comparisons), while the more recent Qwen3.5:9B attains the best open-source portion estimation (NMAE =70 g), a ranking that holds for the NMAE, the cpMAE, and the meal-level error, but not when hallucinated items are also penalized, in which case Qwen2.5VL:7B leads among open-source models. At the primary threshold, the $F_{1}$ score of Qwen2.5VL:7B (0.68) is comparable to those of GPT-5.2 and GPT-5.5 (0.67 and 0.66), although below those of GPT-4o and GPT-4.1.
- A model-specific linear calibration of the portion size predictions reduces the error only for the model with the largest systematic bias (Qwen2.5VL:7B, with cross-validated gains that match the in-sample ones); for the most recent models, whose residual errors are concentrated in the largest portions, it brings no benefit.

We stress that the benchmark evaluates numerical weight estimates generated by the models from single photographs, which should not be equated with a physical measurement of portion size. These results suggest that current general-purpose VLMs require targeted fine-tuning or additional training on food-specific datasets to achieve reliable performance in nutrition-related applications. Future work could explore multimodal datasets with explicit portion-size annotations, incorporate depth or scale references, or integrate external tools, such as 3D estimation models [63], to improve quantity estimation.

Overall, this benchmark highlights both the promise and limitations of existing open-source VLMs in food understanding and establishes a foundation, together with a publicly released and human-validated evaluation protocol, for the development of more specialized models in this domain.

## Figures and Tables

**Figure 1:**
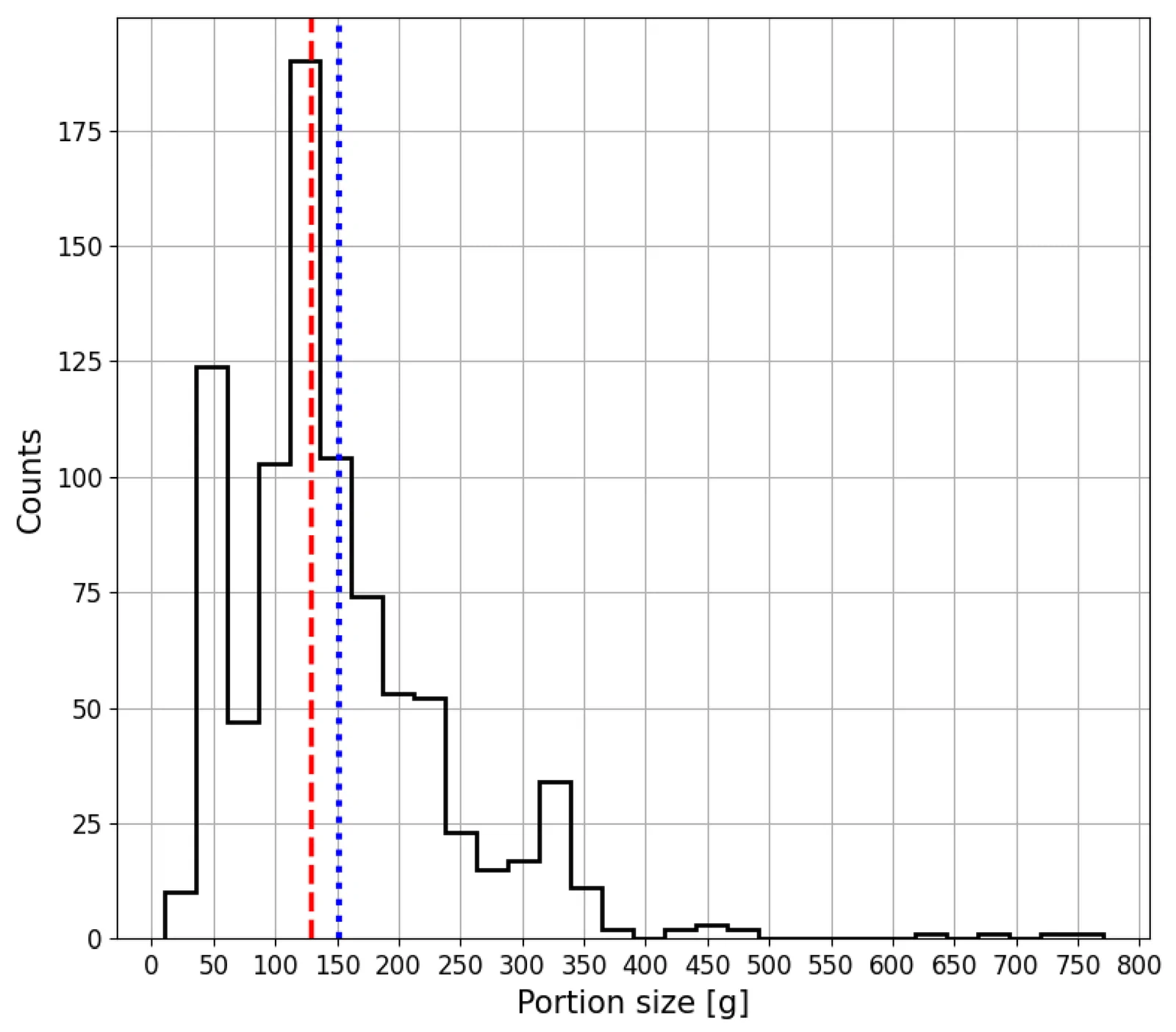
Histogram of the portion sizes (in grams) for the 870 food items in the dataset. The red dashed line corresponds to the median of the distribution, while the blue dotted line corresponds to its mean.

**Figure 2:**
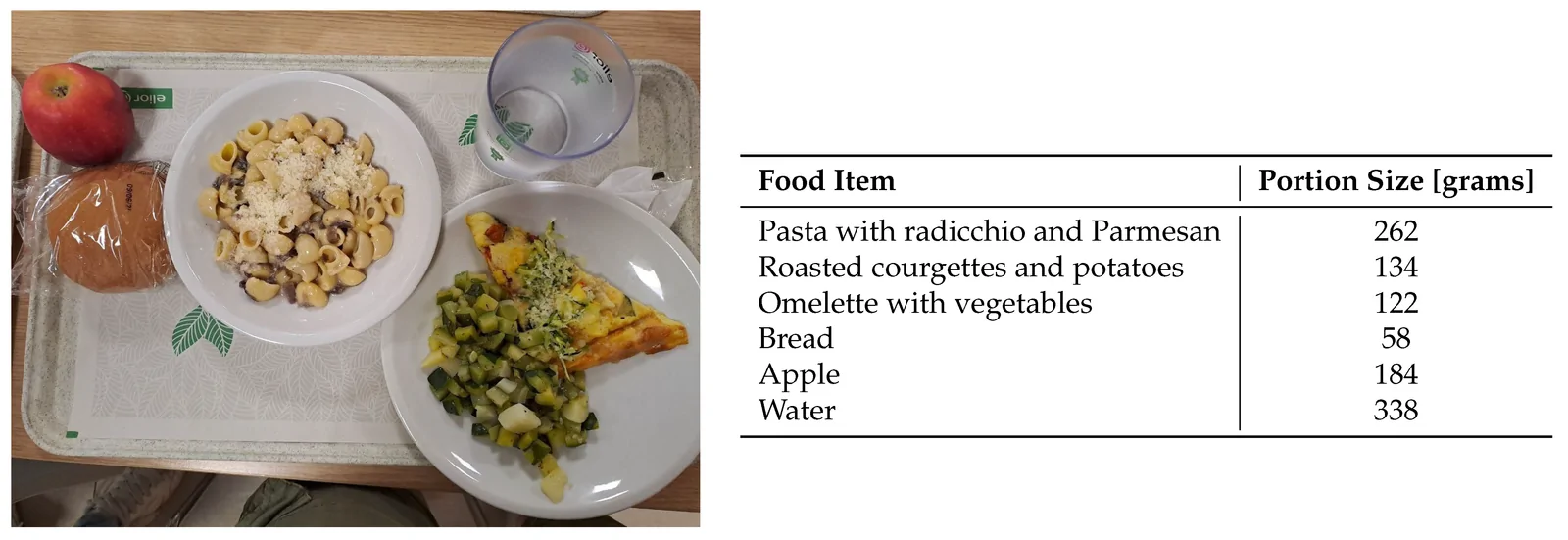
Example food image from the database (**left**) and corresponding ground truth labels (**right**), containing the name of each food item in the image, and the corresponding portion size in grams.

**Figure 3:**
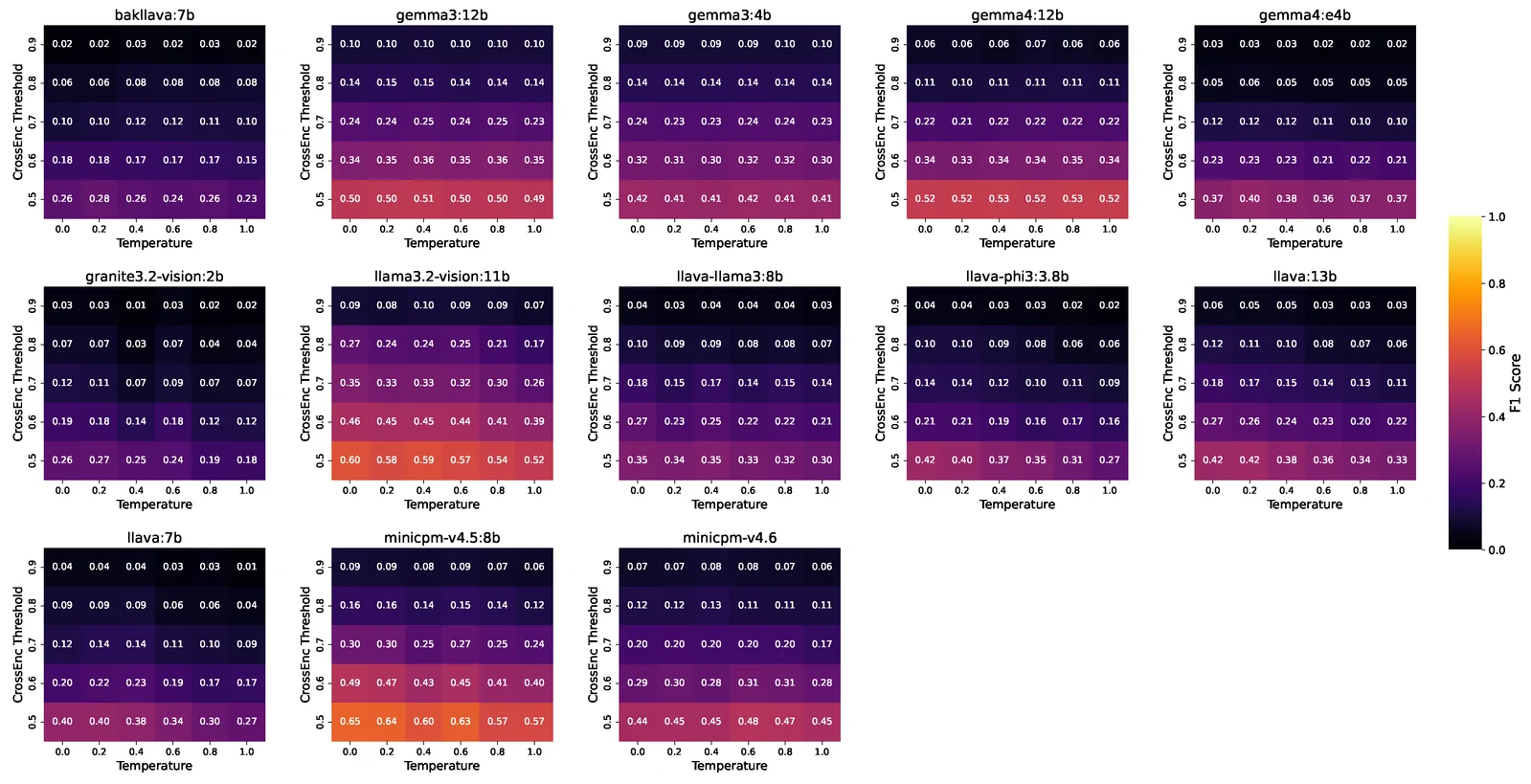
Performance comparison of the open-source VLMs (first half, in alphabetical order). Each panel shows the $F_{1}$ score as a function of model temperature and of Cross Encoder threshold. Continues in Figure 4.

**Figure 4:**
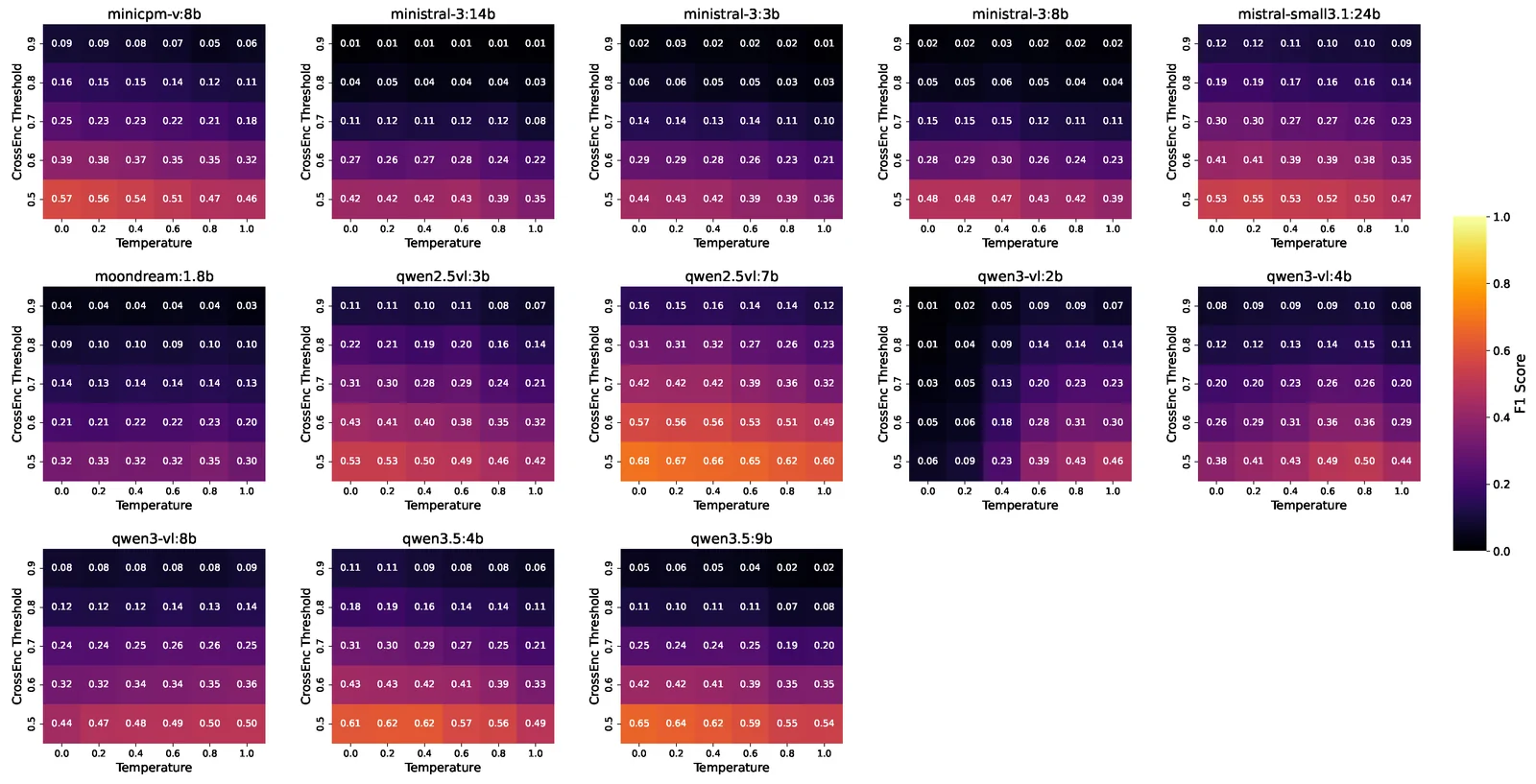
Continuation of Figure 3: $F_{1}$ score for the second half of the open-source VLMs.

**Figure 5:**
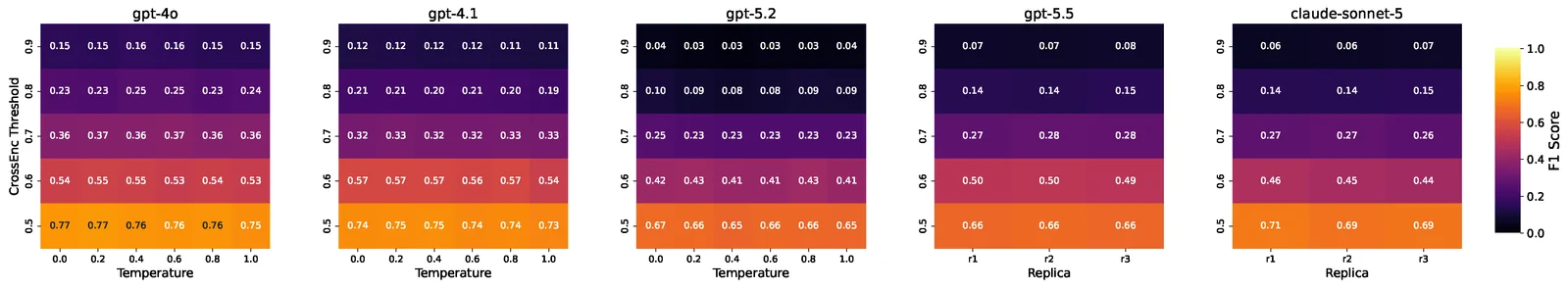
$F_{1}$ score for the five proprietary baseline models. For GPT-4o, GPT-4.1, and GPT-5.2 the horizontal axis is the model temperature; for GPT-5.5 and Claude Sonnet 5, which do not expose a temperature parameter, the horizontal axis reports the three independent replicate runs at the provider default. The near-identical columns of the replicate panels illustrate the small run-to-run variability.

**Figure 6:**
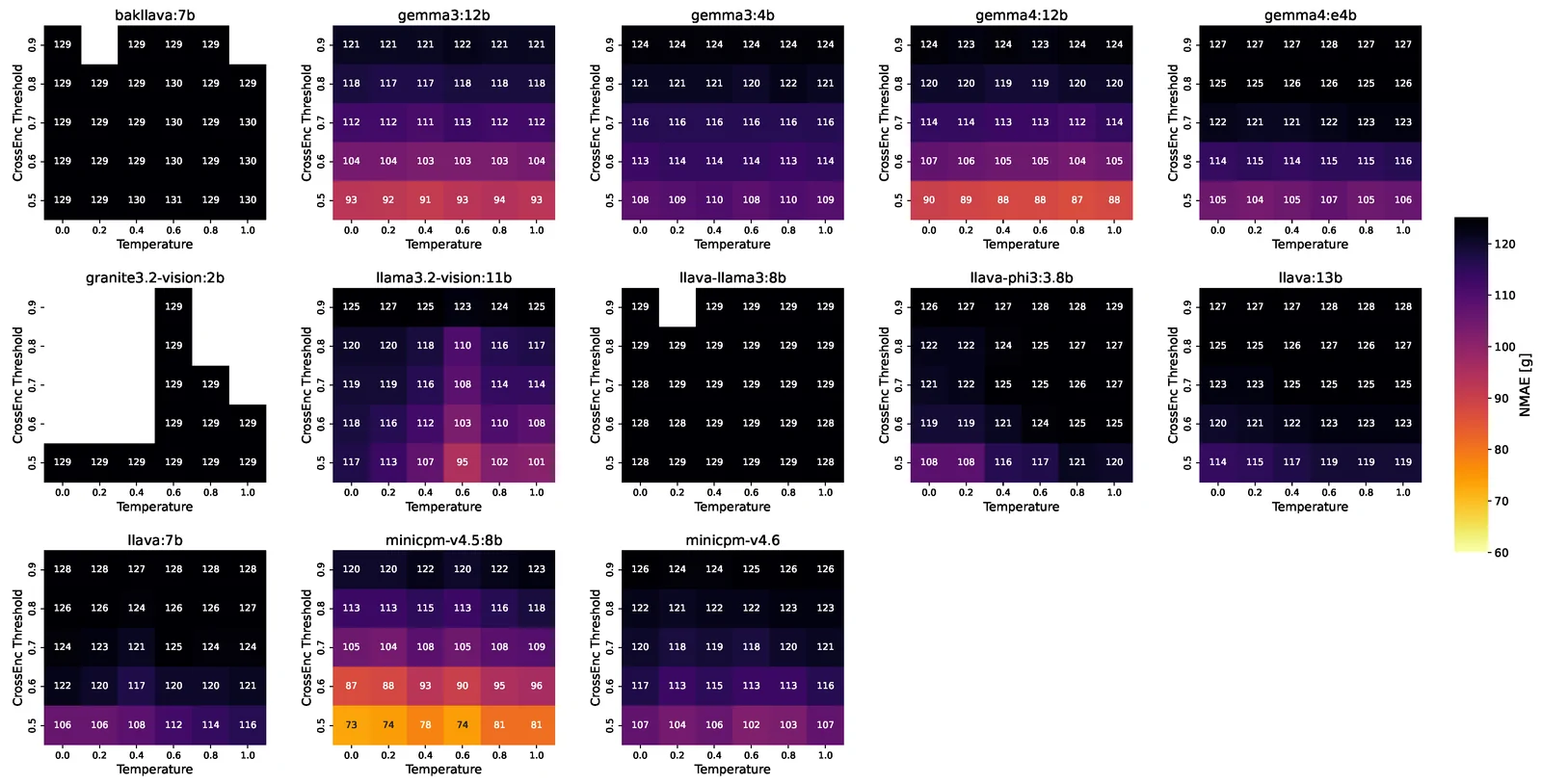
Same as Figure 3, but showing the NMAE (first half of the open-source VLMs). Continues in Figure 7.

**Figure 7:**
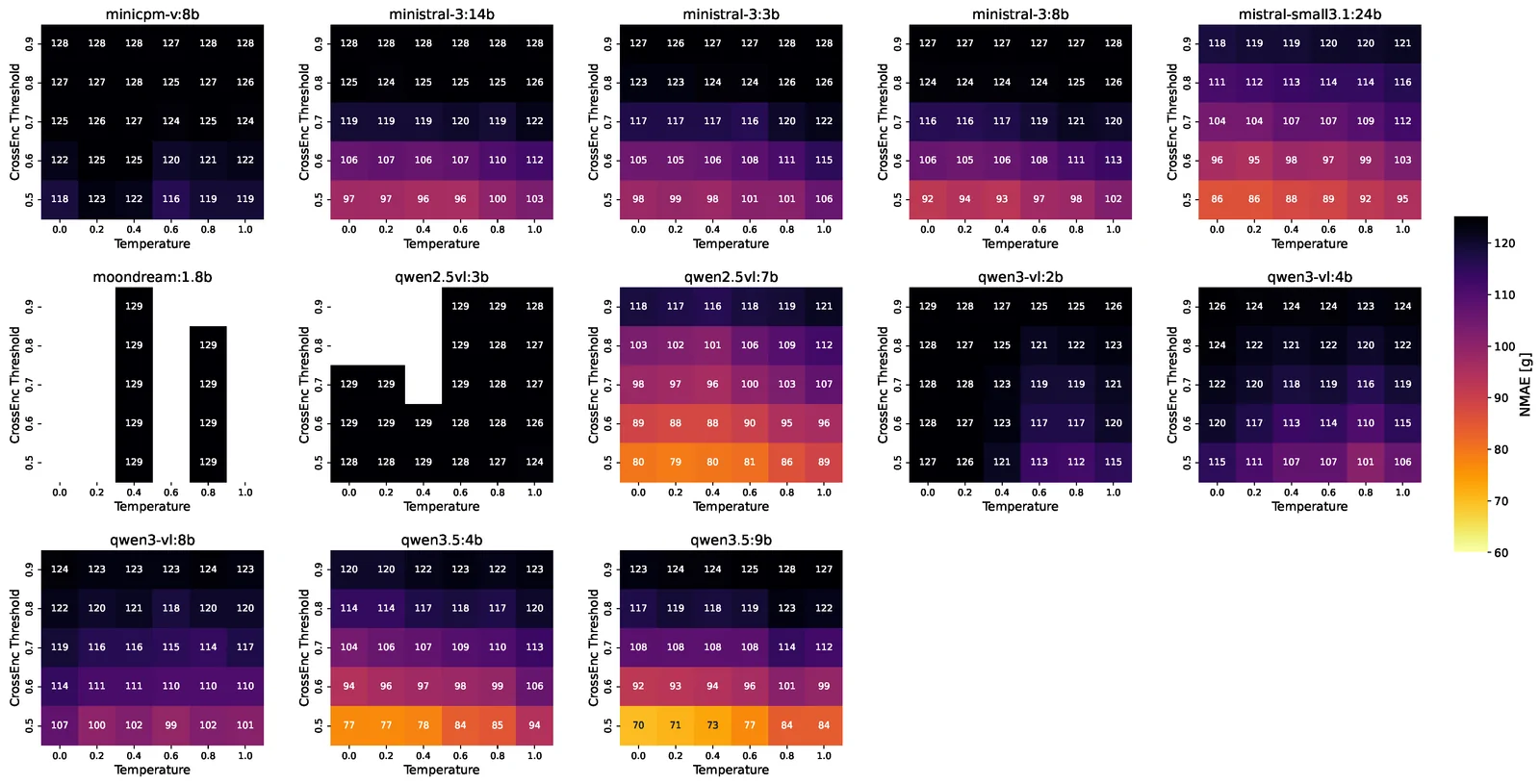
Continuation of Figure 6: NMAE for the second half of the open-source VLMs.

**Figure 8:**
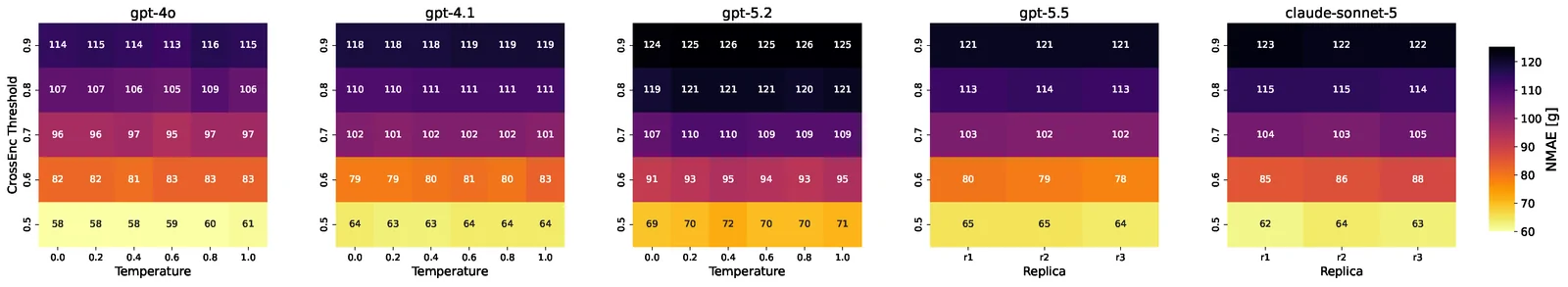
Same as Figure 5, but showing the NMAE for the five proprietary baselines.

**Figure 9:**
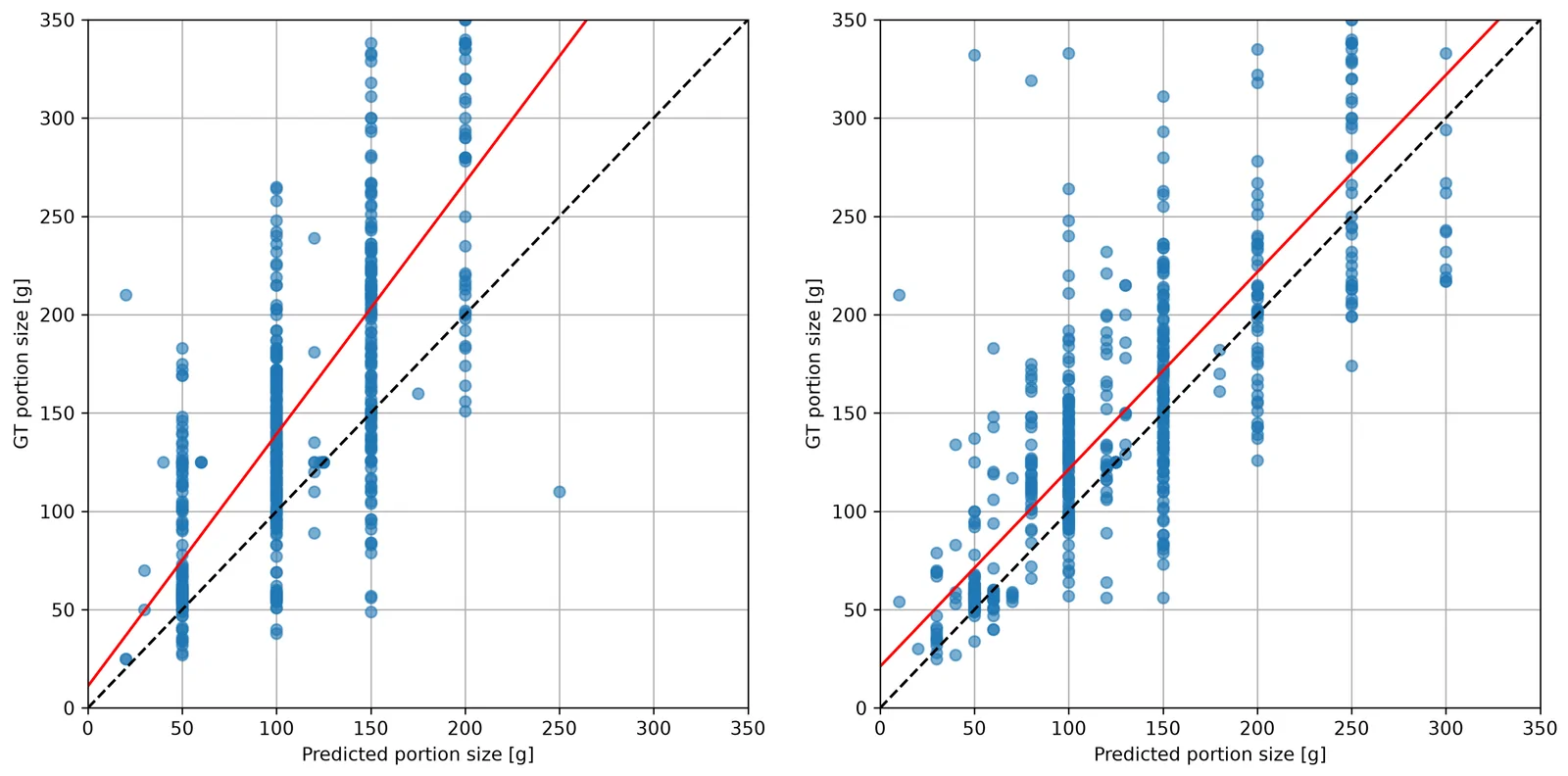
Ground-truth portion size as a function of predicted portion size for the open-source VLM Qwen2.5VL:7B at T=0 and ε=0.5 (**left**) and for the OpenAI GPT-4o model at T=0.2 and ε=0.5 (**right**), i.e., at the configurations of Table 2. The black dashed line is the identity line, while the red solid line is the linear fit to the data.

**Table 1:** Overview of the VLMs evaluated in this study. For each model, we report the number of parameters, model size, and the primary reference describing the model. Some models have multiple configurations; this table lists all configurations explored in this work.

Model Name	Number of Parameters	Model Size (GB)	Reference
BakLLaVA	7B	4.7	[41]
Gemma 3	4B	3.3	[42]
	12B	8.1	
Gemma 4	E4B	9.6	[43]
	12B	7.6	
Granite Vision 3.2	2B	2.4	[44]
Llama 3.2-Vision	11B	7.8	[45,46]
LLaVA	7B	4.7	[47]
	13B	8.0	
LLaVA-Llama 3	8B	5.5	[48]
LLaVA-Phi3	3.8B	2.9	[49]
MiniCPM-V 2.6	8B	5.5	[50,51]
MiniCPM-V 4.5	8B	5.5	[52]
MiniCPM-V 4.6	1.3B	1.3	[53]
Ministral-3	3B	3.0	[54]
	8B	6.0	
	14B	9.1	
Mistral Small 3.1	24B	15	[55]
moondream 2	1.8B	1.7	[56]
Qwen2.5-VL	3B	3.2	[57]
	7B	6.0	
Qwen3-VL	2B	1.9	[58]
	4B	3.3	
	8B	6.1	
Qwen3.5	4B	3.4	[59]
	9B	6.6	

**Table 2:** Summary of the evaluation metrics for all models, at ε=0.5 and at the temperature *T* maximizing the $F_{1}$ score (for GPT-5.5 and Claude Sonnet 5, the first replicate at the provider default). The $F_{1}$ score is reported with its bootstrap 95% confidence interval. *P* is the number of matched items with an associated portion estimate: portion metrics computed on very few items (small *P*) are not reliable. MAE and MedAE are computed on the matched items, while NMAE (Equation (1)) and cpMAE (Equation (2)) also penalize missed items; cpMAE+FP (Equation (3)) additionally penalizes the predicted weight of hallucinated items, and Meal AE (Equation (4)) is the mean absolute error on the total weight of each meal. All portion metrics are in grams. The last three rows report the reference predictors of Section 2.2.4: the typical-serving predictor bounds the portion accuracy achievable from food identity alone, which it is given a priori (it performs no recognition).

Model	*T*	F_1_ [95% CI]	*P*	MAE	MedAE	NMAE	cpMAE	cpMAE+FP	Meal AE
qwen2.5vl:7b	0.0	0.68 [0.66, 0.71]	566	54	40	80	88	113	243
qwen3.5:9b	0.0	0.65 [0.62, 0.68]	582	41	24	70	77	131	118
minicpm-v4.5:8b	0.0	0.65 [0.61, 0.68]	591	47	27	73	80	128	137
qwen3.5:4b	0.4	0.62 [0.59, 0.65]	523	44	30	78	90	140	137
llama3.2-vision:11b	0.0	0.60 [0.57, 0.64]	179	68	49	117	135	150	524
minicpm-v:8b	0.0	0.57 [0.54, 0.60]	128	55	44	118	139	149	535
mistral-small3.1:24b	0.2	0.55 [0.51, 0.58]	464	48	30	86	94	147	167
qwen2.5vl:3b	0.0	0.53 [0.48, 0.57]	5	21	0	128	151	151	658
gemma4:12b	0.4	0.53 [0.49, 0.56]	458	51	33	88	102	170	168
gemma3:12b	0.4	0.51 [0.48, 0.54]	417	50	31	91	103	147	236
qwen3-vl:4b	0.8	0.50 [0.46, 0.55]	310	50	31	101	114	139	386
qwen3-vl:8b	1.0	0.50 [0.46, 0.54]	349	59	34	101	112	151	308
minicpm-v4.6	0.6	0.48 [0.44, 0.52]	372	67	50	102	117	159	320
ministral-3:8b	0.0	0.48 [0.45, 0.51]	393	47	25	92	105	172	167
qwen3-vl:2b	1.0	0.46 [0.42, 0.50]	275	84	66	115	127	147	442
ministral-3:3b	0.0	0.44 [0.40, 0.47]	375	58	39	98	107	166	194
ministral-3:14b	0.6	0.43 [0.40, 0.46]	377	53	34	96	109	182	174
llava:13b	0.2	0.42 [0.39, 0.45]	163	53	42	115	136	152	512
gemma3:4b	0.6	0.42 [0.39, 0.45]	299	69	50	108	115	148	344
llava-phi3:3.8b	0.0	0.42 [0.39, 0.45]	321	72	50	108	125	165	329
llava:7b	0.2	0.40 [0.37, 0.43]	319	66	44	106	121	177	292
gemma4:e4b	0.2	0.40 [0.37, 0.43]	334	65	48	104	122	200	166
llava-llama3:8b	0.4	0.35 [0.32, 0.38]	3	58	77	129	152	153	651
moondream:1.8b	0.8	0.35 [0.32, 0.37]	2	62	62	129	152	152	661
bakllava:7b	0.2	0.28 [0.24, 0.32]	16	114	80	129	151	155	647
granite3.2-vision:2b	0.2	0.27 [0.24, 0.30]	1	540	540	129	152	152	659
GPT-4o	0.2	0.77 [0.75, 0.80]	661	35	19	58	68	96	157
GPT-4.1	0.4	0.75 [0.73, 0.78]	678	44	28	63	70	99	172
Claude Sonnet 5	–	0.71 [0.68, 0.73]	654	39	21	62	70	109	125
GPT-5.2	0.0	0.67 [0.64, 0.70]	624	45	24	69	76	132	120
GPT-5.5	–	0.66 [0.63, 0.69]	641	42	20	65	75	127	122
*Typical-serving predictor*	–	–	870	35	19	–	35	35	98
*Global-median predictor*	–	–	870	61	45	–	61	61	134
*Null predictor*	–	–	0	–	–	129	152	152	661

**Table 3:** Model portion estimates compared with the typical-serving reference on the same items, for the models with at least 450 numerical estimates (configurations of Table 2). *n*: matched items with a numerical estimate; MAE in grams; MedAPE in percent; *r*: within-food correlation between estimated and true weights (foods with at least five matched instances), with its bootstrap 95% CI.

		MAE		MedAPE		
Model	*n*	Model	Typical	Model	Typical	*r* Within Food [95% CI]
GPT-4o	661	35	33	17	12	0.25 [0.09, 0.41]
Claude Sonnet 5	654	39	33	17	13	0.22 [0.09, 0.35]
qwen3.5:9b	582	41	35	19	14	0.13 [0.01, 0.24]
GPT-5.5	641	42	33	19	13	0.08 [−0.15, 0.30]
GPT-4.1	678	44	34	24	13	0.22 [0.07, 0.40]
qwen3.5:4b	523	44	34	22	15	0.14 [0.02, 0.32]
GPT-5.2	624	45	34	21	13	0.19 [0.07, 0.30]
minicpm-v4.5:8b	591	47	35	20	13	0.03 [−0.07, 0.20]
mistral-small3.1:24b	464	48	39	21	16	0.01 [−0.10, 0.20]
gemma4:12b	458	51	33	26	13	0.25 [0.13, 0.39]
qwen2.5vl:7b	566	54	37	29	14	0.07 [−0.03, 0.15]

**Table 4:** Best-fit linear calibration parameters (Equation (5)) and median absolute error (MedAE, grams) before and after calibration, for a representative subset of open-source (top block) and proprietary (bottom block) models at the configurations of Table 2 (ε=0.5). The calibrated MedAE is computed with leave-one-image-out cross-validation. For GPT-5.5 and Claude Sonnet 5, which do not expose a temperature parameter, the fit pools the matched items of the three replicate runs at the provider default temperature.

Model	*T*	*b* [g]	*k*	MedAE Raw	MedAE Calibrated
Qwen2.5VL:7B	0.0	11.1	1.30	40.0	32.5
Qwen3.5:9B	0.0	33.2	0.80	24.5	26.1
MiniCPM-V4.5:8B	0.0	40.6	0.86	27.0	32.0
GPT-4o	0.2	21.1	1.00	19.0	21.6
GPT-4.1	0.4	31.5	0.97	28.0	27.8
GPT-5.2	0.0	38.4	0.84	24.0	31.4
GPT-5.5	–	46.1	0.71	20.0	31.1
Claude Sonnet 5	–	29.8	0.89	20.0	27.1

## Data Availability

All data and code needed to reproduce this study are openly available at https://github.com/Metabolic-Intelligence-Lab/vlm-food-benchmark accessed on 28 October 2026. (code under the MIT license, data under CC BY 4.0).

## Appendix A. Dataset Composition

Table A1 reports the composition of the dataset by food category and by participant. Categories were assigned by keyword rules applied to the ground-truth descriptions (first matching rule wins); the full mapping of the 314 unique descriptions is released with the code.

**Table A1:** Composition of the dataset by food category (top block) and by participant (bottom block). Weights are in grams; entries not applicable to a block are marked with a dash.

Group	Images	Items	Unique Names	Items per Image	Median Weight	Meals
*Food category*						
Bread	154	160	13	–	57.5	–
Pasta, rice and grains	115	116	62	–	213	–
Vegetables	84	97	43	–	129	–
Meat and other protein dishes	80	93	55	–	124	–
Fruit	84	86	13	–	150	–
Desserts and yogurt	65	66	11	–	125	–
Beverages	58	59	4	–	330	–
Potatoes	40	41	20	–	120	–
Soups	38	38	13	–	241.5	–
Legumes	36	36	24	–	129.5	–
Cheese and dairy	34	34	29	–	128	–
Other	28	29	18	–	131	–
Fish	15	15	9	–	112	–
*Participant*						
Participant 1	48	240	–	5.00	125	canteen meals
Participant 2	28	139	–	4.96	125	canteen meals
Participant 3	34	36	–	1.06	268.5	home-prepared lunches
Participant 4	22	112	–	5.09	125.5	canteen meals
Participant 5	8	45	–	5.62	130	canteen meals
Participant 6	60	298	–	4.97	144	canteen meals
**Total**	200	870	314	4.35	129	

## Appendix B. System Prompt

In this appendix, we report the complete system prompt used to instruct the VLMs on their role and objectives. The prompt also elicits a self-reported confidence value for each item; this field was collected but not used in the analysis.

## Appendix C. Accounting of Prediction Outcomes

Table A2 reports, for every model at the configuration depicted in Table 2 (ε=0.5), the number of recognized items with a numerical estimate (*P*), recognized items without a numerical estimate (${\mathrm{TP}}_{⌀}$), missed items (FN), and false-positive predictions (FP), using constructions $P+{\mathrm{TP}}_{⌀}+\mathrm{FN}=870$ and $Q={\mathrm{TP}}_{⌀}+\mathrm{FN}$ defined in Equation (1). The NMAE is reported for three values of the penalty $w_{0}$. The last two columns report the percentage of matches accepted by the Cross Encoder that the LLM judge of Section 2.2.2 considers to be different foods, and the NMAE ($w_{0}=129$ g) recomputed after re-scoring these matches as a missed item plus a false positive.

**Table A2:** Accounting of prediction outcomes and sensitivity of the NMAE (grams) to the penalty $w_{0}$ and to incorrect matches, at ε=0.5.

Model	*P*	TP_⌀_	FN	FP	NMAE, *w*_0_ =			Judged	NMAE
					64.5	129	258	Wrong (%)	(Judge)
qwen2.5vl:7b	566	0	304	219	58	80	125	14	84
qwen3.5:9b	582	0	288	342	49	70	113	13	76
minicpm-v4.5:8b	591	0	279	370	53	73	115	11	76
qwen3.5:4b	523	0	347	300	52	78	129	17	85
llama3.2-vision:11b	179	381	310	431	65	117	219	14	117
minicpm-v:8b	128	334	408	285	63	118	228	22	120
mistral-small3.1:24b	464	21	385	424	56	86	146	20	91
qwen2.5vl:3b	5	422	443	317	64	128	257	19	128
gemma4:12b	458	1	411	418	57	88	149	17	91
gemma3:12b	417	12	441	382	57	91	158	25	96
qwen3-vl:4b	310	75	485	270	59	101	184	13	102
qwen3-vl:8b	349	77	444	410	62	101	178	14	102
minicpm-v4.6	372	1	497	303	65	102	176	12	103
ministral-3:8b	393	43	434	520	57	92	163	25	99
qwen3-vl:2b	275	89	506	345	71	115	203	13	115
ministral-3:3b	375	24	471	562	61	98	172	26	104
ministral-3:14b	377	26	467	602	60	96	169	23	100
llava:13b	163	182	525	411	62	115	220	31	119
gemma3:4b	299	9	562	288	66	108	193	27	112
llava-phi3:3.8b	321	1	548	351	67	108	189	20	111
llava:7b	319	12	539	458	65	106	188	30	112
gemma4:e4b	334	0	536	476	65	104	184	24	108
llava-llama3:8b	3	287	580	487	64	129	257	20	129
moondream:1.8b	2	255	613	354	64	129	258	32	129
bakllava:7b	16	170	684	258	65	129	255	29	129
granite3.2-vision:2b	1	164	705	180	65	129	258	33	129
GPT-4o	661	21	188	209	42	58	89	13	65
GPT-4.1	678	2	190	260	48	63	91	11	67
Claude Sonnet 5	654	4	212	335	46	62	94	12	68
GPT-5.2	624	0	246	370	50	69	105	13	73
GPT-5.5	641	0	229	436	48	65	99	9	65

Table A3 reports, for every model, the share of outputs that were valid JSON, empty, or converted by the repair model, together with the $F_{1}$ score (at the configuration depicted in Table 2) obtained as published, when every repaired output is treated as an empty prediction, and when the repair model is replaced by a deterministic, tolerant JSON parser alone. The three $F_{1}$ scores are computed on a common subset of 116 images, so that they are comparable with one another but differ slightly from those of Table 2.

**Table A3:** JSON failure rates and sensitivity of the $F_{1}$ score (ε=0.5) to the repair step.

	Outputs (%)			F_1_		
Model	Valid	Empty	Repaired	As Published	Repaired → Empty	Deterministic Only
qwen2.5vl:7b	99.9	0.0	0.1	0.69	0.69	0.69
qwen3.5:9b	99.8	0.0	0.2	0.61	0.61	0.61
minicpm-v4.5:8b	100.0	0.0	0.0	0.60	0.60	0.60
qwen3.5:4b	100.0	0.0	0.0	0.59	0.59	0.59
llama3.2-vision:11b	53.2	0.0	46.8	0.58	0.00	0.03
minicpm-v:8b	96.8	0.0	3.2	0.57	0.57	0.57
mistral-small3.1:24b	100.0	0.0	0.0	0.51	0.51	0.51
qwen2.5vl:3b	96.8	0.0	3.2	0.52	0.54	0.54
gemma4:12b	91.4	5.8	2.8	0.51	0.51	0.51
gemma3:12b	100.0	0.0	0.0	0.54	0.54	0.54
qwen3-vl:4b	41.1	32.3	26.6	0.52	0.29	0.32
qwen3-vl:8b	53.4	18.1	28.4	0.53	0.37	0.37
minicpm-v4.6	60.3	29.5	10.2	0.52	0.51	0.51
ministral-3:8b	99.6	0.0	0.4	0.45	0.45	0.45
qwen3-vl:2b	26.1	57.2	16.7	0.46	0.06	0.06
ministral-3:3b	97.0	0.0	3.0	0.43	0.43	0.43
ministral-3:14b	100.0	0.0	0.0	0.41	0.41	0.41
llava:13b	0.0	0.0	100.0	0.39	0.00	0.00
gemma3:4b	100.0	0.0	0.0	0.45	0.45	0.45
llava-phi3:3.8b	85.3	0.0	14.7	0.42	0.40	0.40
llava:7b	87.8	0.0	12.2	0.42	0.41	0.42
gemma4:e4b	100.0	0.0	0.0	0.39	0.39	0.39
llava-llama3:8b	0.0	0.0	100.0	0.35	0.00	0.00
moondream:1.8b	0.0	0.0	100.0	0.34	0.00	0.00
bakllava:7b	18.4	0.0	81.6	0.29	0.00	0.00
granite3.2-vision:2b	0.0	0.0	100.0	0.27	0.00	0.00
GPT-4o	100.0	0.0	0.0	0.77	0.77	0.77
GPT-4.1	100.0	0.0	0.0	0.73	0.73	0.73
Claude Sonnet 5	100.0	0.0	0.0	0.69	0.69	0.69
GPT-5.2	100.0	0.0	0.0	0.65	0.65	0.65
GPT-5.5	100.0	0.0	0.0	0.65	0.65	0.65

## Appendix D. Concise-Name Prompt Experiment

The concise-name prompt is identical to the system prompt of Appendix B, except that the description of the name field reads “shortest common name of the food, as a person would log it (e.g., "orange juice", "grilled chicken", "beef stew")” and that the following constraint is added: “5. Name each food with its shortest common name: do not add cooking details, brands, containers, or other attributes unless they are needed to identify the food.” The three examples do not occur among the ground-truth labels. Each model was run on the 200 images at its configuration of Table 2; no output required JSON repair. Table A4 compares the two prompts; differences are reported with paired image-bootstrap 95% confidence intervals.

**Table A4:** Original versus concise-name prompt (ε=0.5). Words: mean words per predicted name (ground truth: 2.5); items: predicted items per image (ground truth: 4.35); CE: Cross Encoder; Δ: concise minus original.

Model	Prompt	Words	Items	Prec.	Rec.	F_1_ CE	ΔF_1_ CE	F_1_ Judge	ΔF_1_ Judge
GPT-4o	original	3.0	4.5	0.77	0.78	0.77	–	0.72	–
	concise	1.4	4.6	0.70	0.74	0.72	−0.06 [−0.08, −0.03]	0.72	0.00 [−0.03, +0.03]
GPT-5.5	original	4.3	5.4	0.60	0.74	0.66	–	0.66	–
	concise	1.4	5.6	0.58	0.75	0.65	−0.01 [−0.03, +0.02]	0.63	−0.04 [−0.06, −0.01]
qwen2.5vl:7b	original	2.4	3.9	0.72	0.65	0.68	–	0.61	–
	concise	1.3	4.1	0.67	0.64	0.66	−0.03 [−0.06, +0.00]	0.64	+0.03 [−0.01, +0.06]
qwen3.5:9b	original	5.1	4.6	0.63	0.67	0.65	–	0.65	–
	concise	1.5	4.7	0.63	0.68	0.66	+0.01 [−0.03, +0.04]	0.62	−0.03 [−0.05, +0.00]
